# Comparative transcriptome analysis of two contrasting resistant and susceptible *Aegilops tauschii* accessions to wheat leaf rust (*Puccinia triticina*) using RNA-sequencing

**DOI:** 10.1038/s41598-021-04329-x

**Published:** 2022-01-17

**Authors:** Saeideh Dorostkar, Ali Dadkhodaie, Esmaeil Ebrahimie, Bahram Heidari, Mahmood Ahmadi-Kordshooli

**Affiliations:** 1 Department of Plant Production and Genetics, School of Agriculture, Shiraz University, Shiraz, Iran; 2 La Trobe Genomics Research Platform, School of Life Sciences, College of Science, Health and Engineering, La Trobe University, Melbourne, VIC 3086 Australia; 3 School of Animal and Veterinary Sciences, The University of Adelaide, Adelaide, SA 5371 Australia; 4 School of BioSciences, The University of Melbourne, Melbourne, VIC 3052 Australia

**Keywords:** Biological techniques, Biotechnology, Computational biology and bioinformatics, Genetics, Plant sciences

## Abstract

Leaf rust, caused by *Puccinia triticina* Eriks., is the most common rust disease of wheat (*Triticum aestivum* L.) worldwide. Owing to the rapid evolution of virulent pathotypes, new and effective leaf rust resistance sources must be found. *Aegilops tauschii*, an excellent source of resistance genes to a wide range of diseases and pests, may provide novel routes for resistance to this disease. In this study, we aimed to elucidate the transcriptome of leaf rust resistance in two contrasting resistant and susceptible *Ae. tauschii* accessions using RNA-sequencing. Gene ontology, analysis of pathway enrichment and transcription factors provided an apprehensible review of differentially expressed genes and highlighted biological mechanisms behind the *Aegilops*–*P. triticina* interaction. The results showed the resistant accession could uniquely recognize pathogen invasion and respond precisely via reducing galactosyltransferase and overexpressing chromatin remodeling, signaling pathways, cellular homeostasis regulation, alkaloid biosynthesis pathway and alpha-linolenic acid metabolism. However, the suppression of photosynthetic pathway and external stimulus responses were observed upon rust infection in the susceptible genotype. In particular, this first report of comparative transcriptome analysis offers an insight into the strength and weakness of *Aegilops* against leaf rust and exhibits a pipeline for future wheat breeding programs.

## Introduction

Leaf rust (LR), caused by *Puccinia triticina* (*Pt*), is an important wheat disease worldwide^1^. The causal fungus is an obligatory biotrophic pathogen with a complicated life cycle^2,3^ and its spores have the ability to travel long distances by wind. The disease develops rapidly under optimal environmental conditions^4,5^, and the infection spreads to further parasitize wheat, causing yield losses up to 70%^3,6^ resulting from a reduction in kernel number per ear, lower kernel weight, and degradation in grain quality. To avoid yield losses and reduced quality, host resistance is both cost-effective and environmentally safe^7^. Therefore, developing rust-resistant wheat varieties is undoubtedly an important task for feeding the world’s ever-increasing population^8–10^.

The large size and complexity of the wheat genome and also repetitive elements of 75 to 90% have hindered the efforts to better understand its genome and assess its ancestral species for discovering new genes^11^. The diploid wheat relative *Aegilops tauschii* Coss. (DD), as the progenitor of the wheat D genome, is extensively adapted to adverse environmental conditions such as rust diseases^12^. Further, resistance genes from this species could be successfully transferred into wheat^13,14^ through classical approaches. Moreover, because of its smaller genome, it can contribute to the understanding of the wheat genome. Majka et al.^1^ reported that selected accessions of *Ae. tauschii* showed differences in chromosome organization and polymorphism of molecular markers linked to leaf rust and powdery mildew resistance genes, and therefore, could be used to improve wheat and triticale.

RNA-sequencing technology is an important tool in elucidating the molecular mechanisms of resistance to rust, evaluating gene profiles and the transcriptome of different plant organs, introducing candidate resistance genes, and identifying associated markers for marker-assisted breeding^15^. This technology has recently become more affordable to analyze the transcriptomes of both host and pathogen in compatible and incompatible interactions^16–18^. Yadav et al.^18^ reported that the near-isogenic line carrying *Lr57* (WL711 + *Lr57*) had a greater number of differentially expressed genes (DEGs) than the susceptible genotype (WL711). Specifically, more protein kinases and pathogenesis-related (PR) proteins such as chitinases, glucanases were expressed in the resistant genotype. The comparative analysis of these genotypes led to the identification of uniquely expressed transcripts in WL711 + *Lr57*. Also, in a study by Lee et al.^10^, two *Ae. tauschii* accessions, which exhibited hyper-sensitive responses to leaf rust at both seedling and adult plant stages, showed high transcriptional activities of β-1,3-glucanase and peroxidase.

Despite these achievements, a few examples of *Aegilops* whole transcriptome analyses accounting for rust diseases have been conducted^10^; Moreover, no in-depth studies based on both compatible and incompatible host–pathogen interactions are available. Therefore, this study was aimed to analyze the transcriptome of two contrasting leaf rust resistant and susceptible *Aegilops* accessions, and validate the expression of selected genes using RT-qPCR. To our knowledge, this is the first use of the RNA-seq approach to assess the molecular aspects of host resistance and differential gene expression in two Iranian *Ae. tauschii* accessions with compatible and incompatible interactions. The results of this study provide new insights into the molecular mechanisms underlying leaf rust resistance in *Ae. tauschii*, which can aid in resistance breeding strategies.

## Results

### The phenotypic response of *Ae. tauschii *accessions to *P. triticina* at the seedling stage

**Figure 1:**
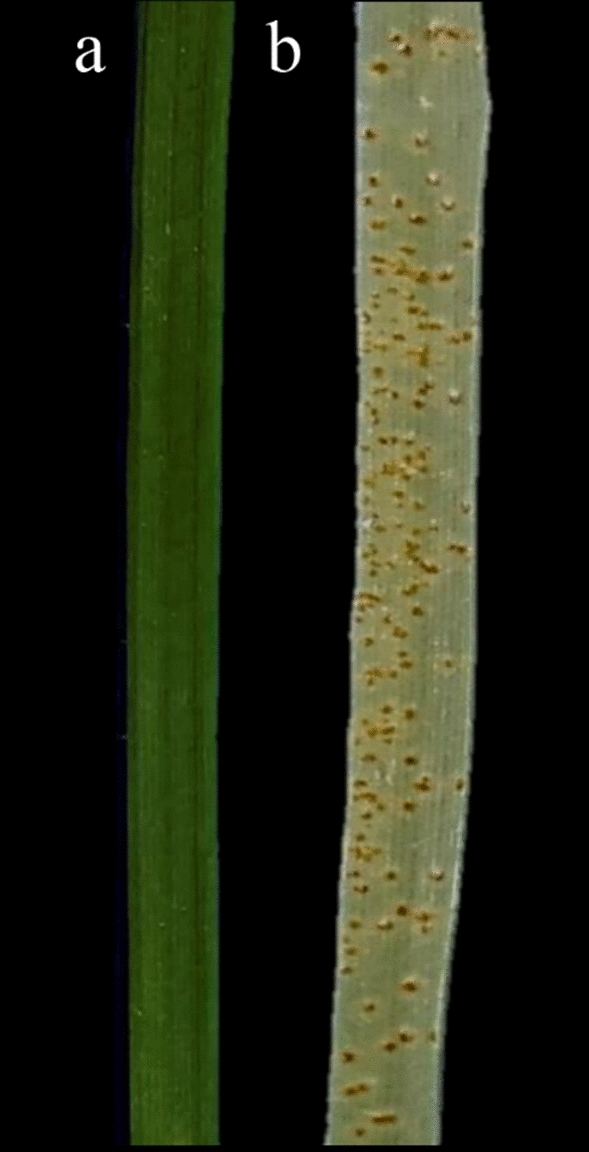
The response of *Aegilops tauschii* accessions AT349 and AT350 to *Puccinia triticina* pathotype CDHLQ at the seedling stage, (**a**) Resistant genotype (IT of ‘0^;=^’), (**b**) Susceptible genotype (IT of ‘3^+^’), Photographed by Saeideh Dorostkar).

The resistant accession of *Ae. tauschii* (AT349) produced an IT of ‘0^;=^’ (hypersensitive flecks) while the susceptible one (AT350) displayed an IT of ‘3^+^’ (large uredinia without surrounding chlorosis; Fig. 1). Both mock-inoculated genotypes used in this study showed no symptoms of infection. Leaf tissues from both inoculated and mock-inoculated plants were sampled 24 h post inoculation (hpi) to monitor transcriptome profiling, and plants from both treatments were maintained for 10–14 days post inoculation until the susceptible cultivar showed a high IT of ‘3^+^’. Based on the phenotyping results, it was clarified that the resistant genotype carries seedling resistance genes and then was used for downstream comparative transcriptome analysis.

### RNA sequencing and mapping statistics

The results of RNA–seq analysis generated a total of 22.76 Gbp end clean data (225.34 × 10^6^ reads) with a length of 101 base pair from the four transcriptome samples including resistant control (RC), resistant treatment (RT), susceptible control (SC) and susceptible treatment (ST). After filtering out rRNA, duplicate sequences, ambiguous and low-quality reads, and *P. triticina* contaminations, an average of 56.55 million high-quality (HQ) clean reads remained per library. The GC percentages with a QC30 base > 95.45%, and the details of the data and their quality before and after filtering are presented in Table 1.

**Table 1:** Sequencing statistics of the four transcriptome samples in two *Ae. tauschii* accessions inoculated with *P. triticina* pathotype CDHLQ at the seedling stage (RT and ST) and mock-inoculated ones (RC and SC).

Library name	RC	RT	SC	ST
Number of raw reads (×10^6^)	71.06	72.05	81.22	56.32
Number of clean reads after the removal of low quality and duplicate reads (×10^6^)	58.37	57.92	66.01	47.23
rRNA clean up (×10^6^)	0.79	0.69	1.00	0.47
*P. triticina* contamination (×10^6^)	0.00	0.95	0.00	0.29
Number of final clean reads (×10^6^)	57.58	56.28	65.01	46.47
GC percentages (%)	54.5	55	55	53

*RC* resistant control, *RT* resistant treatment, *SC* susceptible control, *ST* susceptible treatment.

Then, the HQ clean reads were mapped to the *Ae. tauschii* reference genome (Genome length = 4.2 Gbp including 7 chromosomes). Approximately, 54.39 million clean reads (96.25%) were uniquely mapped while only 2.01% were located in several positions. The remaining 1.73% did not match to the reference genome (Table 2).

**Table 2:** Sequence alignment statistics of the four transcriptome libraries in inoculated *Ae. tauschii* accessions with *P. triticina* pathotype CDHLQ and mock-inoculated ones at the seedling stage with the reference genome (GCA_002575655.1–v.4 from Ensembl).

Library name	RC	RT	SC	ST
Number of HQ clean reads uniquely mapped (×10^6^)	55.31	54.89	62.50	44.87
Uniquely mapped reads (%)	96.16	96.06	96.19	96.54
Multiple mapped reads (%)	1.93	1.97	2.02	2.15
Unmapped reads (%)	1.88	1.96	1.79	1.31
Chimeric reads (%)	0.00	0.00	0.00	0.00

*RC* resistant control, *RT* resistant treatment, *SC* susceptible control, *ST* susceptible treatment.

Subsequently, the principal component analysis (PCA) was carried out to demonstrate differences between samples using their expression profile. Totally, PC1 and PC2 explained 97.9% of variance (Fig. S1). The vicinity of the inoculated and mock-inoculated resistant accession (RT and RC) samples suggests that they have less difference compared to the susceptible ones.

### Validation of RNA-seq data

**Figure 2:**
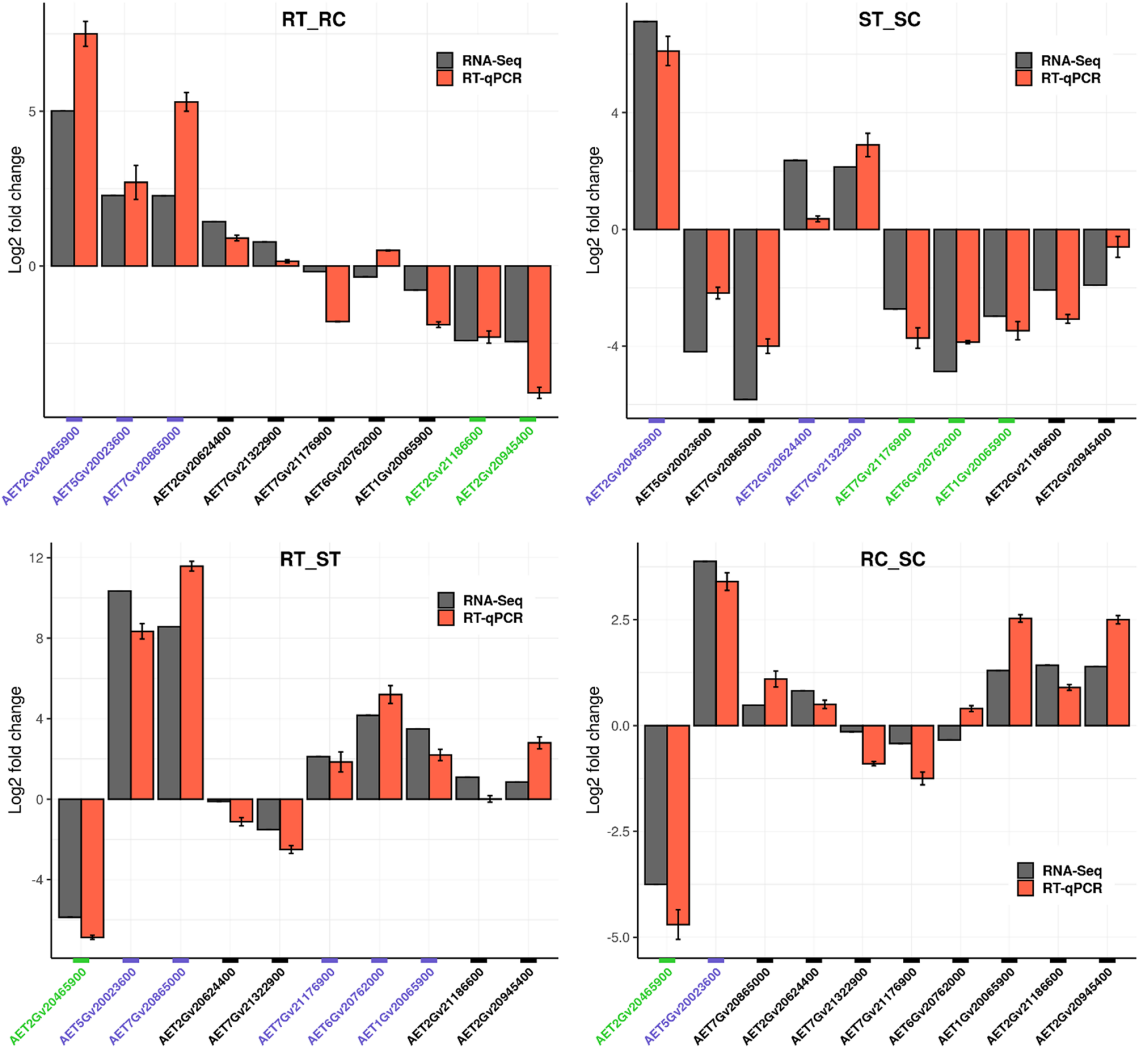
Illustration of the RT-qPCR confirming the results of 10 selected DEGs. The X axis represents the genes and the Y axis indicates the log_2_ (fold-change) values derived from RNA-seq and RT-qPCR in all four comparisons. Purple, green and black colors show up-regulated, down-regulated and non-significant DEGs, respectively. RC (resistant control), RT (resistant treatment), SC (susceptible control), and ST (susceptible treatment). The bar plots were created using ‘ggplot2 version 3.3.5’ R/CRAN package (https://ggplot2.tidyverse.org).

**Figure 3:**
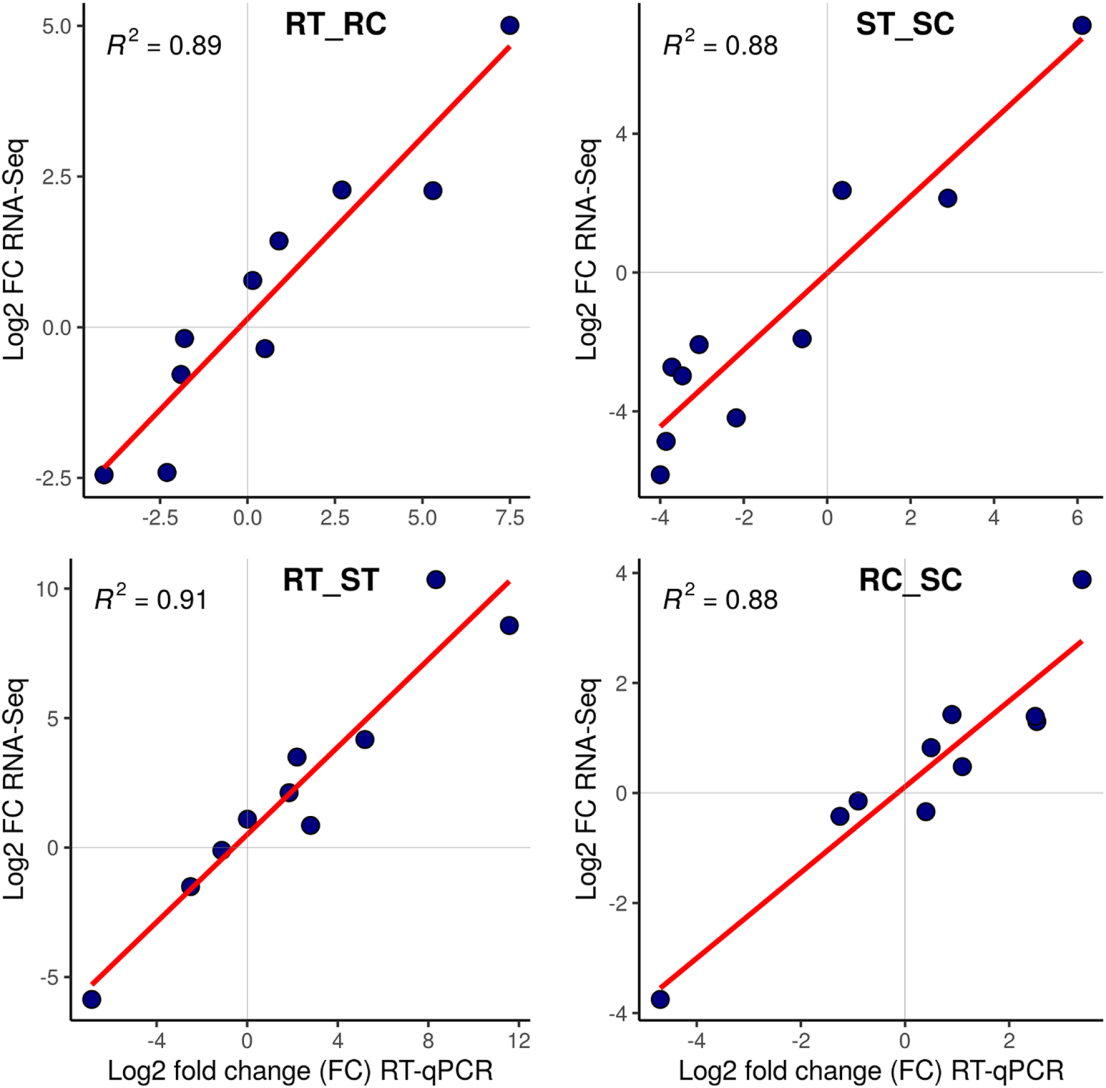
Regression analysis of the log_2_ (fold-change) values between RNA-seq and RT-qPCR in all four comparisons. RC (resistant control), RT (resistant treatment), SC (susceptible control), and ST (susceptible treatment). The plots were created using ‘ggplot2 version 3.3.5’ R/CRAN package (https://ggplot2.tidyverse.org).

Differential expression of each gene was obtained by its estimated ‘probability’ in the NOISeq pipeline where a gene with the probability above 0.95 was considered a DEG. In the present study, DEGs with the highest estimated probability in each experimental condition were selected to assess the validity of the RNA-seq data using RT-qPCR. Furthermore, these DEGs contributed to processes like; oxidation–reduction, photosynthesis, membrane transport, regulation of transcription, peptidase activity and catalase activity. All selected DEGs showed concordant expression patterns with the RT-qPCR results (Fig. 2). There were high positive correlations (R^2^ = 0.89, 0.88, 0.91, and 0.88) between the RNA-seq and RT-qPCR fold changes in the RT_RC, ST_SC, RT_ST, and RC_SC comparisons, respectively (Fig. 3).

### Overall view of differentially expressed genes (DEGs)

**Figure 4:**
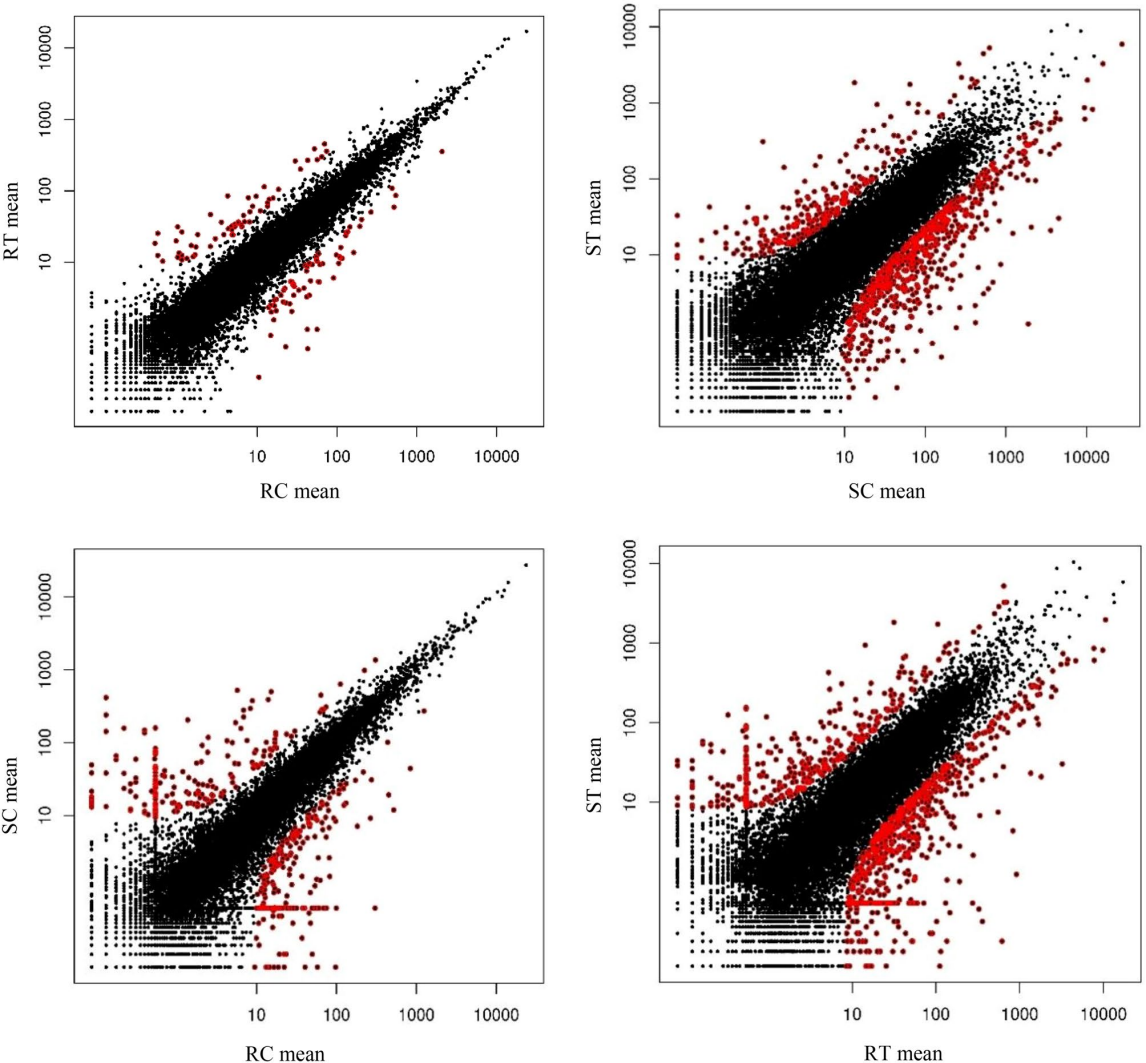
Gene expression scatter plots summarize the expression values for the susceptible and resistant accessions of *Ae. tauschii* under rust inoculation or mock inoculation. The *P. triticina* pathotype CDHLQ was used to inoculate genotypes while the inoculum for mock-inoculated plants included only talcum powder. Expressed genes for both conditions are highlighted in black while differentially expressed genes are shown in red. NOISeq simulated five technical replications for any of the experimental conditions and therefore, X and Y axes represent the average expression values of each condition. RC (resistant control), RT (resistant treatment), SC (susceptible control), and ST (susceptible treatment). The scatter plots were created using the ‘NOISeq’ R/Bioconductor package^20^.

Genes with differential expression probability values higher than 0.95 were considered DEGs as suggested by Tarazona et al.^19^. The total number of reference genome genes was 39,630, of which 26,523 (≈ 66.93%) were differentially expressed among the four samples, and were reduced to 19,145 after filtering out low count reads. As illustrated in the scatter plot (Fig. 4), the results of deep sequencing revealed that a substantial number of DEGs were detected in comparisons that involved the infected susceptible genotype.

**Figure 5:**
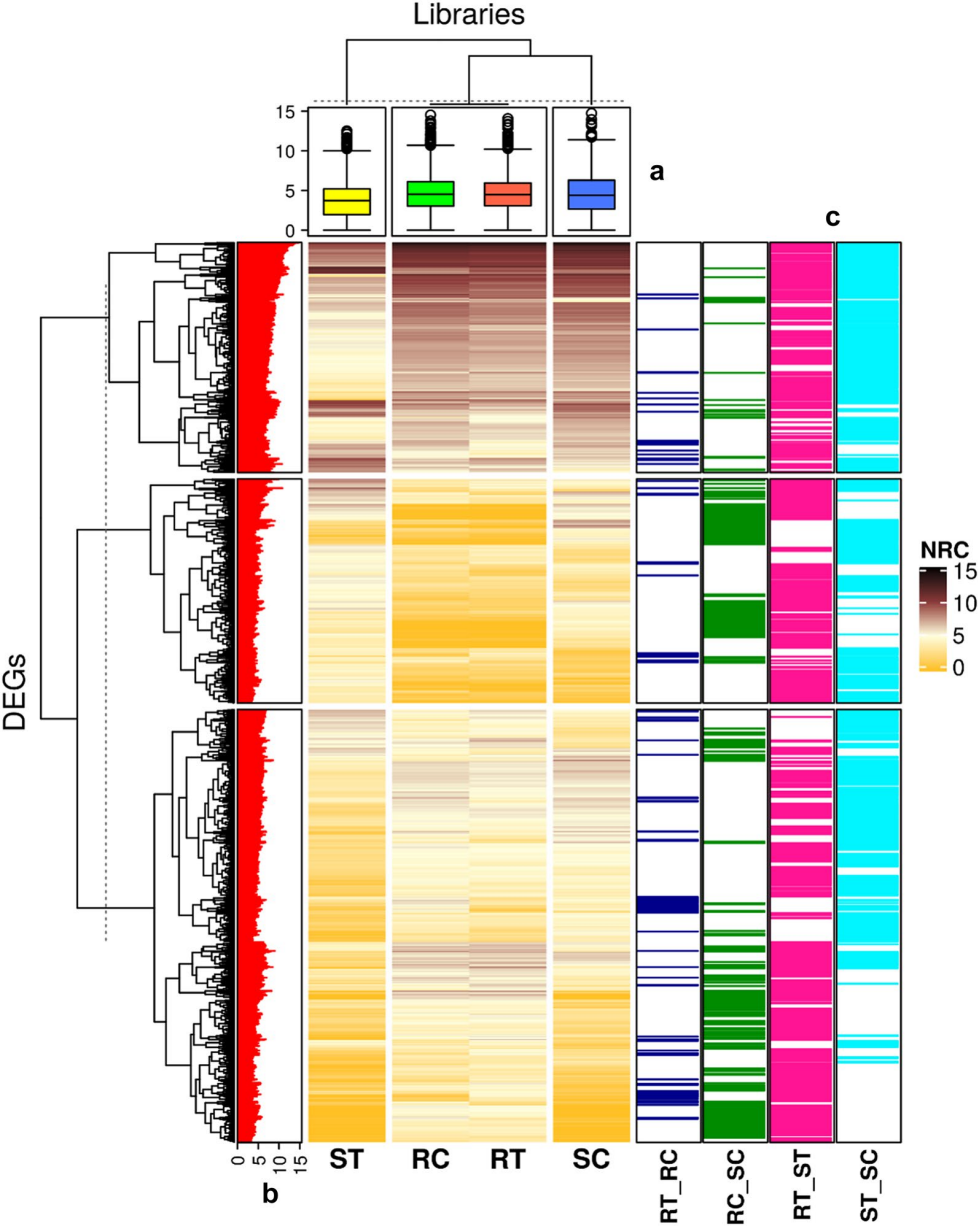
Heat map for log_2_ gene expression profile in the resistant and susceptible genotypes combining with box plot (**a**) and bar plots (**b**,**c**). The rows of main plot represent the differentially expressed genes (DEGs) and columns denote samples. RC (resistant control), RT (resistant treatment, at 24 hpi), SC (susceptible control), and ST (susceptible treatment, at 24 hpi). NRC denotes normalized read count by TMM method. (**a**) The box plot displays distribution of log_2_ gene expression profile. (**b**) The bar plot shows maximum expression of each gene in samples. (**c**) The bar plots denote the frequency of DEGs in each comparison (RT_RC: 120 DEGs, RC_SC: 390 DEGs, RT_ST: 984 DEGs and ST_SC: 905 DEGs). The figure was created using the ‘ComplexHeatmap’ R/Bioconductor package^21^.

Hierarchical clustering for gene expression profiles revealed the presence of three major clusters based on samples (libraries) (Fig. 5a) or DEGs (Fig. 5b). Cluster I consisted of both RC and RT libraries while the SC and ST libraries were separated into two different clusters (Fig. 5a). Although the gene expression profiles of the RC and RT samples were similar, the ST library showed a completely different profile. In other words, a high number of DEGs was identified in response to leaf rust in comparisons that involved ST (i.e., RT_ST and ST_SC) while the RT_RC comparison represented a small number of DEGs (Fig. 5c).

**Figure 6:**
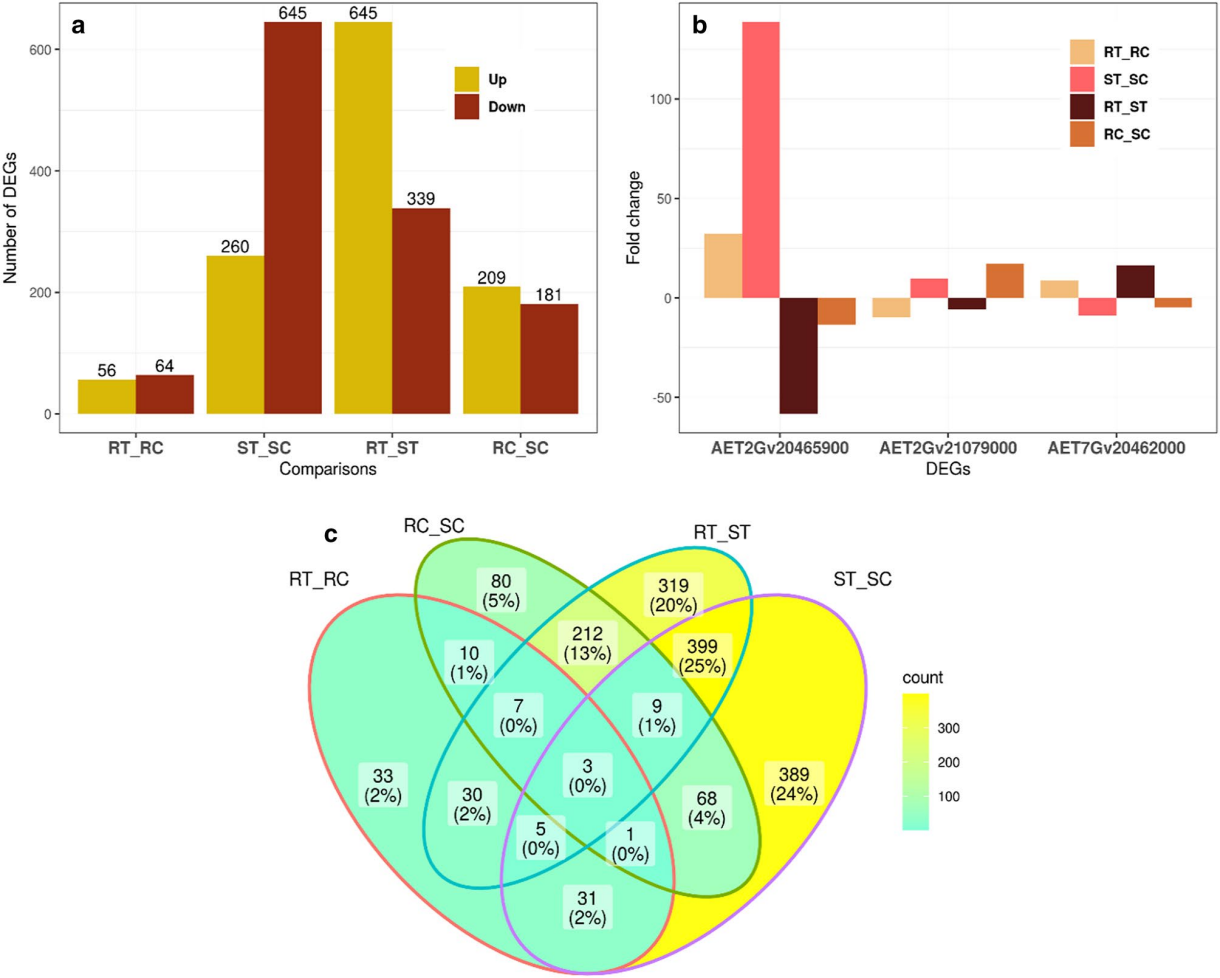
Summary of differentially expressed genes (DEGs) in four comparisons. RC (resistant control), RT (resistant treatment), SC (susceptible control), and ST (susceptible treatment). (**a**) Up and down-regulated DEG statistics in different comparisons of the resistant or susceptible *Ae. tauschii* accessions (AT349 or AT350) with/out *P. triticina* infection, (**b**) Fold-changes of three common DEGs among all comparisons. (**c**) The Venn diagram exhibits the distribution of DEGs in different comparisons. 6a and 6b were created using ‘ggplot2 version 3.3.5’ R/CRAN package https://ggplot2.tidyverse.org) while, 6c was created using ‘ggVennDiagram version 1.2.0’ R/CRAN package (https://github.com/gaospecial/ggVennDiagram).

Under the control conditions of RC_SC, 390 genes were differentially expressed, of which 209 were up-regulated and 181 were down-regulated. In the presence of leaf rust infection (24 hpi), 984 DEGs were detected in RT_ST, of which 645 were up-regulated while the remaining 339 genes were down-regulated. The number of DEGs in the RT_ST comparison was higher than that of RC_SC indicating the effect of leaf rust on gene expression. For RT_RC, 120 genes (56 up-regulated and 64 down-regulated) were differentially expressed, while the value was 905 (260 up-regulated and 645 down-regulated) for ST_SC (Figs. 5c and 6a). The results showed three gene IDs including ‘*AET2Gv20465900*’ (chromosome 2D), ‘*AET7Gv20462000*’ (chromosome 7D) and ‘*AET2Gv21079000*’ (chromosome 2D) were common among all comparisons (Figs. 6b,c).

### Gene ontology overview of DEGs

**Figure 7:**
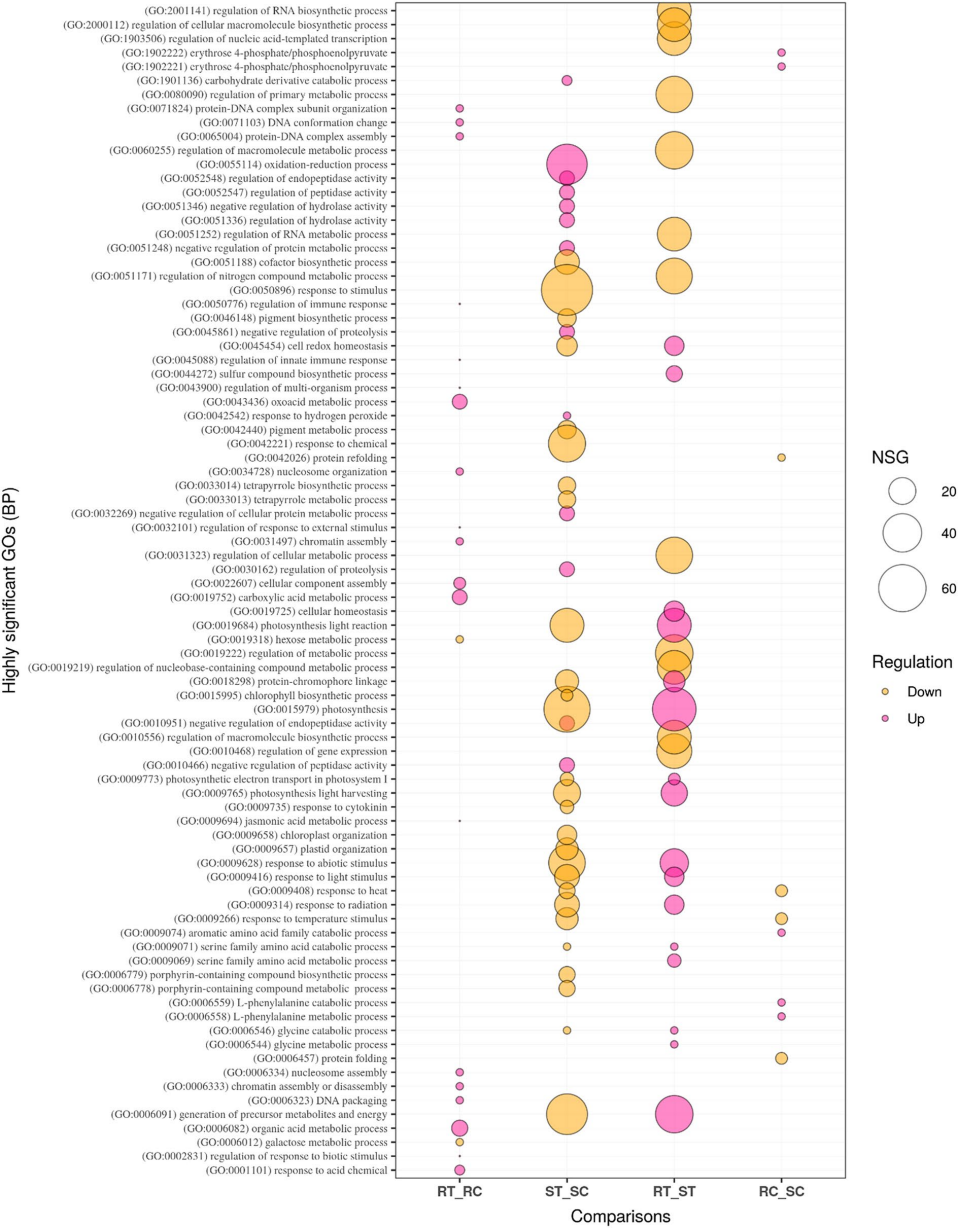
Bubble plot of the GO enrichment terms related to biological process (BP). The size of the circles shows the number of significant genes (NSG) associated with each GO term. Purple and orange colors show up-regulated and down-regulated DEGs, respectively. RC (resistant control), RT (resistant treatment), SC (susceptible control), and ST (susceptible treatment). The plot was created using ‘ggplot2 version 3.3.5’ R/CRAN package (https://ggplot2.tidyverse.org).

**Figure 8:**
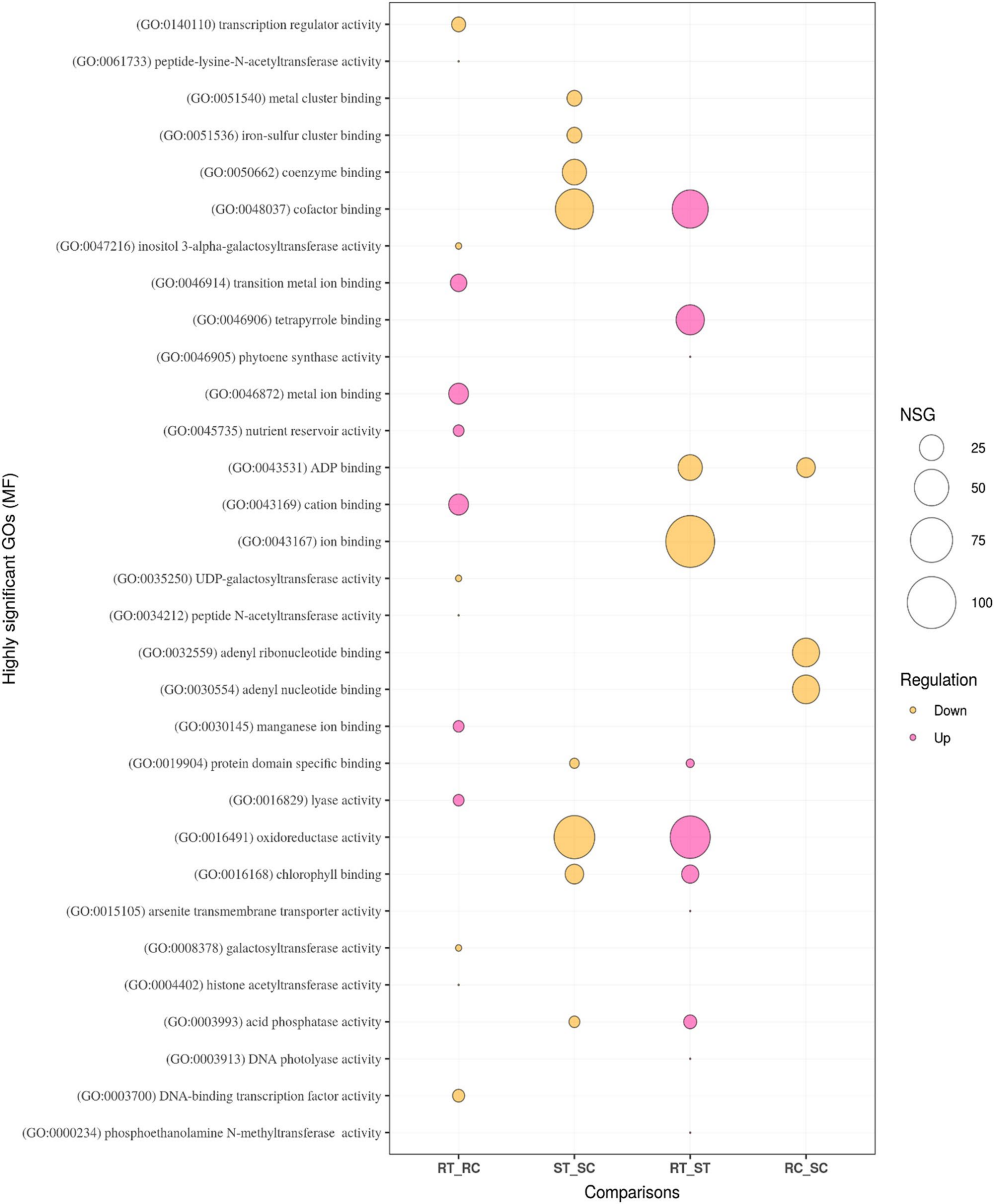
Bubble plot of the GO enrichment terms related to molecular function (MF). The size of the circles shows the number of significant genes (NSG) associated with each GO term. Purple and orange colors show up-regulated and down-regulated DEGs, respectively. RC (resistant control), RT (resistant treatment), SC (susceptible control), and ST (susceptible treatment). The plot was created using ‘ggplot2 version 3.3.5’ R/CRAN package (https://ggplot2.tidyverse.org).

Gene ontology enrichment analysis of biological process (BP), molecular function (MF), and cellular component (CC) was performed to further examine the DEGs in the four comparisons. Eighty GO terms related to the BP category, 40 related to the MF category, and eight related to CC were identified in the RT_RC comparison that outlined the resistance mechanism of the AT349 accession. Of the GO terms in BP, a large number of the up-regulated DEGs in the inoculated resistant accession were associated with the significantly enriched GO terms like; ‘organic acid, carboxylic acid and oxoacid metabolic processes’, ‘response to acid chemical’, ‘cellular component assembly’ as well as the Go terms related to ‘nucleosome and chromatin organization’. Moreover, a small number of the up-regulated DEGs coordinated with ‘regulation of response to biotic and external stimulus’, ‘jasmonic acid metabolic process’, ‘regulation of multi-organism process’ and ‘immune response and regulation of innate immune response’. ‘Galactose and hexose metabolic processes’ were highly significantly enriched GO terms related to the down-regulated DEGs (Fig. 7). With regards to MF, the most abundant terms were ‘ion binding’, ‘nutrient reservoir activity’, ‘lyase activity’ and ‘acetyltransfrase activity’ in the up-regulated DEGs while ‘galactosyltransferase activity’, ‘transcription regulator activity’ and ‘DNA-binding transcription factor activity’ constituted the most frequent terms in the down-regulated DEGs (Fig. 8). The ‘extracellular region’, ‘nucleosome’ and ‘chromatin’ ranked first to third in the up-regulated DEGs related to the CC category (Fig. S2).

Gene ontology exploration within ST_SC explained the susceptibility responses of AT350 to leaf rust. The number of GO terms related to the BP, MF, and CC categories were 162, 92, and 11, respectively. From the window of BP, the most powerful GO terms including ‘photosynthesis’, ‘response to stimulus’, ‘generation of precursor metabolites and energy’, ‘porphyrin-containing compound’ and ‘tetrapyrole metabolic and biosynthetic process’, ‘pigment and cofactor biosynthetic processes’, ‘response to abiotic stimulus like heat, temperature, radiation and light’, ‘plastid and chloroplast organization’ were relevant to the down-regulated DEGs in the inoculated susceptible accession; AT350. The DEGs associated with negative regulation (which stops, prevents or reduces the frequency, rate or extent of some processes) of GO terms like ‘proteolysis’, ‘hydrolase’, ‘cellular protein’, ‘peptidase and endopeptidase’ were up-regulated. Also, the other terms like; ‘hydrolase activity’, ‘oxidation–reduction process’ and ‘response to hydrogen peroxide’ were the highly significant GO terms associated with the up-regulated DEGs (Fig. 7). The top GO terms of MF category were ‘peptidase inhibitor activity’ and ‘endo peptidase inhibitor activity’ in the up-regulated DEGs and ‘chlorophyll binding’, ‘cofactor binding’ and ‘protein domain specific binding’ in the down-regulated ones in this comparison (Fig. 8). In the CC category, ‘membrane’ was the most frequently detected term in the up-regulated DEGs while ‘stromule’ and ‘photosystem I reaction center’ were frequent terms in the down-regulated DEGs (Fig. S2).

The responses of the inoculated AT349 and AT350 accessions against rust infection were described in comparing RT and ST. The BP GO terms like; ‘generation of precursor of metabolites and energy’, ‘serine family amino acid catabolic and metabolic processes’, ‘glycine catabolic and metabolic processes’, ‘response to abiotic stimulus, radiation and light’, ‘cellular homeostasis’, ‘cell redox homeostasis’, ‘photosynthesis process’ together with ‘sulfur compound biosynthetic process’ were associated with the up-regulated DEGs in the inoculated AT349 contrary to AT350. However, the down-regulated DEGs were integrated with regulation processes including ‘gene expression’, ‘macromolecule biosynthetic’, ‘cellular metabolic’, ‘primary metabolic’, ‘RNA biosynthetic metabolic’ and ‘regulation of nucleic acid-templated transcription’ (Fig. 7). In terms of MF, the down-regulated DEGs were associated with ‘ion binding’ while the up-regulated DEGs were related to ‘chlorophyll binding’, ‘acid phosphatase activity’, and ‘cofactor binding’ (Fig. 8). In the CC category, a large number of the up-regulated DEGs in RT related to the ‘membrane’ and ‘intracellular anatomical structure’ (Fig. S2).

The natural differences between the resistant and susceptible accessions were obtained from a comparison between mock-inoculated RC and SC represented by the AT349 and AT350 accessions, respectively. The DEGs associated with BP processes like; ‘L-phenylalanine metabolic and catabolic’, ‘aromatic amino acid catabolic’ as well as ‘phosphoenolpyruvate metabolic and catabolic’ were up-regulated in the mock-inoculated AT349 while those associated with ‘responses to heat and temperature stimulus’ as well as ‘protein folding and refolding’ were down-regulated (Fig. 7). Regarding MF, the down-regulated DEGs related to the ‘nucleotide binding’, ‘chaperon binding’, ‘purine binding’ and ‘lyse activity’ while the up-regulated DEGs associated with ‘acid phosphatase, ribonuclease and hydrolase activities’ (Fig. 8 and Fig. S3). The GO term ‘Sec61 translocon complex’ was up-regulated in the CC category (Fig. S2). More details ‘on biological processes, molecular functions, and cellular components as well as the number of genes associated with each GO term are presented in Figs. 7 and 8, and Supplementary Fig. S2.

### DEGs through the lens of pathway enrichment analysis

Pathway enrichment analysis in the RT_RC comparison suggested that the up-regulated DEGs in the inoculated AT349 accession were involved in ‘alpha-linolenic acid and linoleic acid metabolism’, ‘alkaloid biosynthesis’, ‘nitrogen metabolism’, ‘glycine, serine, threonine, phenylalanine and tryptophan metabolism’ and ‘selenocompound metabolism’, in contrast to the mock-inoculated AT349. ‘Galactose metabolism’ pathway was associated with the down-regulated DEGs in this comparison.

‘Photosynthesis’, ‘glyoxylate and dicarboxylate metabolism’, ‘glycolysis/gluconeogenesis’ and ‘protein processing in endoplasmic reticulum (ER)’ were the most common enriched metabolic pathways in the down-regulated DEGs of the ST_SC comparison. The signaling pathways including ‘NOD-like receptor signaling pathway’, and ‘plant hormone signal transduction’ were the other down-regulated pathways.

The down-regulated pathways in the ST_SC comparison mentioned above, were up-regulated in RT_ST. In addition, the exclusive pathways ‘glycerolipid metabolism’, ‘one carbon pool by folate’, ‘C–type lectin receptor signaling pathway’, ‘plant–pathogen interaction’, mitogen-activated protein kinases (MAPK) signaling pathway’, ‘calcium signaling pathway’ and ‘RNA degradation’ were up-regulated in AT349 in comparison to AT350 (the RT_ST comparison). Conversely, ‘cell cycle’ pathway was down-regulated. Interestingly, in RC_SC, ‘MAPK signaling pathway’, ‘phenylalanine metabolism’, ‘phenylpropanoid biosynthesis’, ‘amino sugar and nucleotide sugar metabolism’ were up-regulated. Protein processing’ was the only pathway down-regulated in this comparison.

### Querying DEGs to identify transcription factors (TFs) and leucine rich repeat (LRR) proteins

Transcription factors are proteins that bind to the regulatory sequences of DNA molecules triggering decreased or increased gene transcription and consequently regulate gene expression^22^. An up-regulated DEG which encodes ‘basic helix-loop-helix’ (bHLH) transcription factor was identified in the inoculated compared with the mock-inoculated resistant accession (AT349; the RT_RC comparison). The DEGs encoding ‘myeloblastosis-related’ (MYB-related) and ‘Lesions simulating disease resistance’ (LSD) TFs were up and down-regulated in ST_SC, respectively. DEGs encoding TF Ethylene Responsive Factor superfamily consisted of ERF and RAV, ‘N-acetylcysteine’ (NAC), ‘far-red-impaired response’ (FAR1), ‘Nuclear Factor Y’ (NF-YB), WRKY, B3, ‘Basic leucine zipper’ (bZIP), ‘(zinc-finger reverse transcription) (zf-RVT) and ‘Heat Shock Factor’ (HSF) were identified in the RT_ST group, of which only the last two were up-regulated. No TF was observed in the resistant genotype compared to the susceptible one under control conditions.

Based on the importance of the RT_RC and RT_ST comparisons, the NB-LRR and/or LRR motif-containing genes were identified among the DEGs of these two comparisons as listed in Supplementary Table S1. The results showed that two (both up-regulated) and 43 (29 up-regulated and 14 down-regulated) LRR motif-containing genes were detected in the RT_RC and RT_ST comparisons, respectively. The up-regulated ‘*AET5Gv20023600*’ and ‘*AET2Gv20853300*’ DEGs carried an LRR motif in the RT_RC comparison. From the 29 up-regulated LRR-containing genes in the RT_ST comparison, the gene ID ‘*AET3Gv2061130029*’ contained two LRR-motifs while the rest (28 DEGs) had one LRR-motif. Among the down-regulated DEGs in the RT_ST comparison, the ‘*AET5Gv21162800*’ was the only identified gene that encoded both nucleotide-binding (NB-ARC) and leucine-rich domains. Moreover, reciprocal blast was conducted in BLASTN to identify the putative orthologues for available sequences of LR resistance genes in *T. aestivum* (bread wheat). The putative orthologues of the genes *Lr1, 22a, 34* and *67* were found in the *Ae. tauschii* reference genome (Supplementary Table S2).

## Discussion

High-performance gene expression approaches could shed light on the molecular mechanisms of host–pathogen interactions. In particular, next-generation sequencing can be applied to study important non-model host–pathogen systems, such as wheat-*Puccinia* to develop more effective strategies for investigating destructive pathogens^23^. *Ae. tauschii* as a gene resource and the most important diploid progenitor of wheat could greatly benefit disease resistance programs. Phenotyping of *Ae. tauschii* showed low and high ITs of ‘0;^=^’ and ‘3^+^’ for the accessions AT349 and AT350, respectively, which provided the opportunity to comparatively analyze their complete transcriptome and study the differential gene expression. A greater number of DEGs were either up or down-regulated in the ST_SC comparison group than in the RT_RC comparison suggesting that the susceptible genotype was highly disturbed at the transcriptional level. The hyper-responsivity of the susceptible genotype at the molecular level has also been reported in other plant species under biotic^24^ and abiotic stresses^25^. Downstream evaluations including GO enrichment and pathway analysis together with the study of TFs provided a comprehensive review of DEGs in the four comparisons. In addition, the comparison of RT-qPCR results with those of RNA-seq validated the data. Though the expression levels of the selected DEGs using RT-qPCR were slightly different  from the RNA seq data, they showed highly significant correlations, confirming the findings of transcriptome analysis.

### RT_RC comparison

The comparison of the results of the inoculated and mock-inoculated AT349 opened up a window to understand the resistance mechanisms and clarify the molecular aspects of its strategy to precisely respond to rust infection, which is reflected as an IT of ‘0^;=^’. The results revealed that the ‘α-linolenic acid metabolism’ pathway was dynamically overexpressed in the inoculated AT349. In particular, this pathway is associated with jasmonic acid (JA) biosynthesis which is an important signaling molecule closely related to plant defense and resistance to pathogens^26^. The LR pathogen secretes an arsenal of effector proteins interfering with the defense system and potentially could facilitate its colonization. As a result, the host activates an interconnected network of mechanisms to prevent disease progression. In this context, the host JA-mediated immunity from the up-regulated ‘*AET6Gv20822700*’ (6.23-fold) and ‘*AET7Gv21052200*’genes (4.57-fold) encoded allene oxide cyclase and peptidase family, respectively, and activated JA dependent signaling cascades which in turn resulted in regulating the innate immune response. This is in agreement with the results obtained by Berens et al.^26^ and Han and Kahmann^27^ who stated JA biosynthesis leads to the enhancement of plant immunity and resistance.

Likewise, JA pathways can be regulated by chromatin-remodeling factors that affect plant defense^28^. The up-regulated genes ‘*AET5Gv21027500*’ (4.57-fold), ‘*AET5Gv21027300*’ (5.76-fold), and ‘*AET5Gv21029100*’ (4.99-fold) encoding histone H1 are associated with chromatin assembly/disassembly. Histone H1 binds to the nucleosome core and protects the linker DNA between nucleosomes, causes further compaction, and participates in higher-order chromatin structure formation and remodeling^29^. Consequently, chromatin remodeling is a potential means to control gene expression and regulate response to LR pathogen, which is consistent with the study by Alvarez et al.^29^ who stated that defense-related genes are often involved in chromatin modifications and remodeling.

Eight up-regulated DEGs were identified in the metabolic processes of carboxylic acid, oxoacid, and organic acids in the infected AT349 genotype. Carboxylic acid acts as a natural fungicide and decreases the destructive effects of fungal attacks^30,31^. Similarly, organic acids and oxoacids have been reported to protect plants against pathogen infections^32,33^. The functions of these genes were annotated in ion binding, cyclase, cyanase, jasmonyl-hydrolase, and lyase activity. In addition, they processed the metabolism of acids through fatty acid and amino acid metabolism, nitrogen metabolism, and alkaloid biosynthesis pathways. In this context, the aromatic amino acid (phenylalanine, tyrosine, and tryptophan) pathway was significantly activated in RT_ RC compared to ST_SC in response to infection. Aromatic amino acid (AAA) biosynthesis and degradation act as a starting point for the metabolism of a large variety of secondary metabolites that play essential roles in the plant immune system^34^. The genes ‘*AET1Gv20127900*’ (6.47-fold) and ‘*AET2Gv20740100*’ (6.39-fold) encoding pyridoxal–dependent decarboxylase conserved domain (Pyridoxal_deC), and glutamine amidotransferase class-I, respectively, were associated with AAA pathway to biosynthesize secondary metabolites in defense against LR pathogen. Moreover, the resistant genotype (AT349) could uniquely recognize microbial elicitors by receptor proteins and respond to them via the ‘alkaloid biosynthesis’ pathway where the Pyridoxal_deC protein is encoded by ‘*AET1Gv20127900*’. The enzymatic decarboxylation of tyrosine and tryptophan leads to the synthesis of alkaloid products. Alkaloids are often elevated in response to infection^34,35^ and hence, their biosynthesis pathway may have contributed substantially to the AT349’s response to leaf rust. Further, the gene ‘*AET4Gv20718500*’ (6.93-fold) encoding cyanase protein associated with the ‘nitrogen metabolism’ pathway was activated in the infected AT349. This pathway creates essential signals for regulating the responses of plants to environmental changes via glutamine metabolism and arginine biosynthesis, and is consistent with the findings of Fagard et al.^36^ who suggested that glutamine plays an essential role in plant defense responses through the nitrogen metabolism pathway.

Rapid and sophisticated responses via differential gene expression to various environmental factors have evolved in plants by phenomena largely controlled by TFs^37^. The bHLH encoding TF, ‘*AET5Gv20888400*’ (5.37-fold) was the only up-regulated TF found in the inoculated AT349. This TF responds to resistance inducers such as jasmonic acid, which influences defense pathways and alters plant responses from compatibility to incompatibility in cooperation with defense genes^38,39^. Therefore, it can be suggested as a candidate regulator in the resistant accession under biotic stress conditions.

The DEGs ‘*AET2Gv20184700*’ (− 38.32-fold), ‘*AET2Gv20184800*’ (− 5.13-fold), and ‘*AET7Gv21151800*’ (− 74.83-fold), which are associated with hexose sugars specifically galactose metabolism, were down-regulated in the infected AT349. In fact, fungal invasion resulted in a dramatic decrease in GalT enzyme activation in AT349 which catalyzes the attachment of galactose to proteins during glycoprotein synthesis^40^ and has multiple functions in host–pathogen interactions. Despite playing a crucial role in controlling pathogen infection, host glycoproteins may function in opposite ways and promote pathogen infection in some cases. Interestingly, pathogens sometimes secrete non-glycoproteins in the host and glycosylate them using host’s glycosylation facilities to become pathogenic^41^. Depending on the type of host–pathogen system, either a decrease or an increase in sugar level has been observed in infected tissues^42^. Remarkably, the down-regulation of GaIT expression in AT349 may suggest a possible mechanism of pathogen inhibition by limiting the activation of effectors via galactosylation.

### ST_SC comparison

The findings from the ST_SC group explained the circumstances that led to the susceptibility response and defined weaknesses and AT350’s failure against rust. In the infected AT350 accession, down-regulation of a substantial number of DEGs mostly related to the ‘photosynthesis system’, ‘precursor metabolites’, ‘cell energy generation’, and ‘external stimulus responses’ were associated with the susceptible phenotype, and hence an IT of ‘3^+^’ was detected. Importantly, the down-regulation of many influential photosynthesis-annotated genes such as ‘*AET2Gv20124700*’ (− 15.57-fold) encoding ribulose-1,5-bisphosphate carboxylase-oxygenase (RuBisCo) enzyme, ‘*AET6Gv20414400*’ (− 6.56-fold), ‘*AET6Gv20245800*’ (− 195.2-fold) and ‘*AET4Gv20184600*’ (− 7.78-fold) which encode chlorophyll A-B binding proteins, ‘*AET4Gv20128500*’ (− 5.78-fold) encoding photosystem I protein (PSI-N), ‘*AET1Gv20789200*’ (− 9.48-fold) encoding photosystem II protein (PsbW), ‘*AET2Gv20439100*’ (− 5.16-fold) encoding cytochrome B6-F and ‘*AET1Gv20253000*’ (− 5.52-fold) encoding nicotinamide adenine dinucleotide phosphate (NAD(P)H) dehydrogenase may be a result of leaf rust on altered regulation of photosynthesis in the accession AT350. The study of these DEGs could aid in understanding the role of photosynthesis apparatus in plant immunity. This is consistent with the findings of Poretti et al.^24^, Ghosh et al.^43^, and Cohen and Leach^44^, who stated that photosynthesis as a hub of cross-talk in growth and defense trade-offs during plant–pathogen interactions is inhibited by a range of biotic stresses, including bacterial, viral, and fungal pathogens. A considerable number of DEGs which associated with the host energy generation system via precursor metabolic process, were down-regulated in the susceptible accession upon infection. The precursor metabolites are intermediate molecules in the catabolic and anabolic pathways that can be oxidized to generate ATP and supply cell energy or can be used to synthesize macromolecular subunits such as amino acids, lipids, and nucleotides^45^. In addition, responses to various types of stimuli declined by down-regulating a substantial number of related genes in the infected AT350. For instance, a dramatic down-regulation of the ‘*AET1Gv20670800*’ gene (− 21.94-fold) encoding LSD transcription factor was associated with susceptible phenotype in the infected AT350. Moreover, DEGs involved in signaling pathways like ‘NOD-like receptor signaling pathway’, ‘AMPK signaling pathway’ and ‘plant hormone signal transduction’ were significantly knocked down in this genotype. The gene ‘*AET5Gv20169800*’ (− 13.20-fold) encoding leucine-rich repeat (LRR) protein was one of the highly down-regulated genes associated with the ‘hormone signal transduction’ pathway. LRR proteins have a proven role in plant resistance to pathogens^46–48^.

Seven up-regulated DEGs were identified in association with negative regulation (a process that stops or reduces the rate of an activity) of proteolysis, peptidase, endopeptidase, and hydrolase activities, of them ‘*AET5Gv21082200*’ which encodes proteinase inhibitor showed a 331.66-fold change. In fact, proteases and protease inhibitors protect plants against pathogens, and hence their negative regulation results in the inhibition of defense responses. Likewise, studies have reported that down-regulating the genes encoding proteolytic enzymes enhances the susceptibility of plants to pathogens^49^. It proposes that the down-regulation rate of protease activity in the extracellular region of the infected AT350 probably leads to the protection of *P. triticina*’s effector proteins against the host proteolytic machinery, and may suggest the possible role of the proteolytic machinery in defense responses.

### RT_ST comparison

Hidden molecular aspects of the genotype AT349 displaying resistance to rust were evaluated in comparison with the susceptible accession of AT350. The exclusive output of this comparison revealed that ‘serine family amino acid metabolic process’, and ‘glycine metabolic process’ were up-regulated in the inoculated AT349 compared to AT350. Glycine and serine are integrated with photorespiration, which is involved in defense responses during plant–pathogen interactions^50,51^. The aminomethyl transferase folate-binding domain encoding gene; ‘*AET2Gv21032800*’ (5.68-fold) which was identified in the serine/glycine metabolic process, was up-regulated across the ‘one-carbon pool by folate’ pathway. Folates, also known as B9 vitamins, have an overlooked role in plant responses to stresses and innate immunity^52^. Furthermore, ‘sulfur compound biosynthesis’ may have been augmented in AT349, where the up-regulated ‘HSF’ TF gene ‘*AET4Gv20678400*’ (6.93-fold) was involved. Sulfur biosynthesis plays an important role in plant immunity, and is an essential nutrient for metabolite synthesis^53^. This element also acts as a phytoanticipin or phytoalexin to protect plants from various pathogens and pests^53^. Likewise, the up-regulated ‘zf-RVT’ TF (‘*AET5Gv20218800*’ (12.36-fold)) in AT349 might participate in initiating the transcription of downstream defense-related genes or act as potential effector decoys^54^ and therefore, is worthy of further investigation. Furthermore, ‘cellular homeostasis’ was significantly enhanced by up-regulating 12 genes functionally responsible for cofactor binding, oxidoreductase, and catalytic activities in the inoculated AT349, which is critical for maintaining the balance of cell’s biological processes. From the perspective of RNA degradation mechanisms, the gene ‘*AET3Gv20765000*’ which encodes ‘enolase, C-terminal TIM barrel domain’, increased by more than 104 folds in the infected leaves. This domain with lyase and catalytic activities in the phosphopyruvate hydratase complex, possibly leads to the generation of precursor metabolites and energy, as well as a defense response to leaf rust.

The ‘*AET4Gv20478300*’ gene encoding EF domain protein which is involved in calmodulin/calmodulin-like protein (CaM/CLM), was up-regulated by 8.22-folds. CaM/CLM, as a calcium sensor protein, plays a crucial role in cellular signaling cascades and is involved in stomatal closure, cell wall reinforcement, and hypersensitive response^55^. This gene is associated with the ‘plant–pathogen interaction’ pathway probably via enhancing calcium (Ca^2+^) concentration in the cytosol. Further, the afore-mentioned DEG’s association with ‘C–type lectin receptor signaling’ pathway suggests its participation in the perception of pathogen invasion. β-Glucan is one of the most prominent polysaccharides of the fungal cell wall^56^, which initiates the defense response through this pathway. This gene was associated with CaM which in turn, as a member of the signaling pathway may cause cell differentiation and protect it against leaf rust pathogen. Furthermore, this gene is integrated with ‘MAPK signaling’ pathway via a calmodulin protein (CaM4) which induces the activation of mitogen-activated protein kinase8 (MAPK8), promoting the negative regulation of ROS accumulation and maintaining cell homeostasis. These results comport with current models of ETI by Wang et al.^57^, who reported that plants have evolved two types of immune systems (PAMP-triggered immunity (PTI) and effector-triggered immunity (ETI)) to recognize and fend off pathogens via the activation of calcium-dependent protein kinases (CDPKs), prompted ROS burst and activated MAPKs. In the transcriptome study of wheat near-isogenic lines with/out *Lr28*, the resistant plants displayed higher levels of expression in genes encoding MAPK during incompatible interactions at 12–24 hpi as compared to the susceptible ones during compatible interactions^58^. Also, Tang et al.^59^ stated calcium influx activates MAPK cascades which in turn promote stomatal closure to limit the pathogen’s entry into leaves. Furthermore, the ‘*AET1Gv20702500*’ gene encoding UTP-glucose-1-phosphate uridylyltransferase (UDPGP), was up-regulated in the ‘glycerolipid metabolism’ pathway, which is expected to mediate disease resistance responses^60^. Therefore, these genes may be implicated in resistance, supporting their role in *Ae. tauschii* immunity and could serve as potential candidates in future studies.

Interestingly, DEGs involved in ‘regulation of primary and macromolecule metabolic processes’, ‘regulation of nitrogen compound metabolism’, ‘RNA metabolism’, and ‘nucleobase-containing compound metabolic processes’, which result in final ‘regulation of cellular transcription’, were down-regulated within the Rb-EF2 complex of AT349 compared to AT350 upon infection (Fig. S4). The retinoblastoma (Rb) protein, known as retinoblastoma-related (RBR) protein in plants, controls the cell cycle through interacting with the EF2 transcription factor family. In fact, the Rb-EF2 complex plays a crucial role in controlling the transition from G1 to S phase in the plant cell cycle^61^. Reducing cellular transcription associated with the Rb-EF2 complex in the inoculated AT349 probably suggests that AT349 prefers to maintain the cell’s energy against the pathogen via enduring the G1 phase and postponing the S phase and cell division. In addition, the gene ‘*AET7Gv20648500*’ (− 7.23-fold) encoding retinoblastoma-associated protein A domain (Rb-A) associated with the ‘cell cycle’ pathway was down-regulated. Furthermore, a significant number of DEGs encoding transcription factors such as ethylene-responsive factor superfamily consisted of ‘ERF’ and ‘RAV’, ‘NAC’, ‘FAR1’, ‘NF-YB’, ‘WRKY’, ‘B3’ and ‘bZIP’ were associated with the above-mentioned down-regulated processes. Generally, TF responses to unfavorable conditions are complex and their functions are specific and can vary depending on the race of the pathogen^54^.

### RC_SC comparison

The RC_SC comparison revealed differences between the resistant and susceptible accessions in the absence of infection which could be used as criteria for discriminating between these genotypes. The up-regulated DEGs were precisely tracked across the biological processes and pathways in AT349, and ‘*AET1Gv20256300*’ (11.10-fold), ‘*AET2Gv20412400*’ (10.86-fold) and ‘*AET2Gv20412700*’ (8.72-fold) participated in ‘phosphoenolpyruvate and erythrose 4-phosphate metabolism’ process. This is the precursor for the synthesis of aromatic amino acids and also mediates the amino sugar and nucleotide sugar metabolism pathways. These DEGs also integrated into Phe catabolic/metabolic processes where Phe is a building block of many compounds that in turn, mediate plant reproduction, growth, development, and defense against different stresses.

### Monitoring leaf rust (LR) resistance genes and NB-LRR domains

So far, ~ 80 LR resistance genes have been identified and characterized in wheat^62^; some of which were deployed in wheat cultivars. Among them, the genes *Lr21*, *Lr22a*, *Lr32*, *Lr39*/*Lr41, Lr40*, *Lr42*, and *Lr43* have been transferred from *Ae. tauschii* into wheat^63^, of which *Lr21* (GenBank: FJ876280.1) and *Lr22a* (KY064064.1) have been cloned and sequenced^64,65^. Among these two sequenced LR genes, we could identify a putative orthologue gene for *Lr22a* i.e. ‘*AET2Gv20074800*’ with the ATP binding function in *Ae. tauschii* reference genome with 98% identity. However, this putative gene did not appear in DEGs. The main reason for this could be that *Lr22a* is an adult plant resistance (APR) gene^66^ and our samples were taken at the seedling stage. Moreover, four bread wheat-originated LRs i.e. *Lr1* (EF439840.1)^67^, *Lr10* (AY270157.1)^68^, *Lr34* (FJ436983.1)^69^, *Lr67* (MK425206.1)^70^ that have been sequenced, were identified in the *Ae. tauschii* reference genome (Supplementary Table S2). Interestingly, the putative orthologues of *Lr1* (all stage-resistance ‘*AET5Gv21241000*’ with 98% identity), *Lr34* (APR ‘*AET7Gv20224900*’ with 100% identity) and *Lr67* (APR ‘*AET4Gv20606400*’ with 99% identity) appeared in DEGs. Ling et al.^71^ screened 200 accessions of *Ae. tauschii* against *P. triticina* isolates at the seedling stage, and detected and mapped a dominant resistance gene in accession Tr.t. 213 at the same chromosomal position as *Lr1*. They concluded that the resistance gene in this accession is an orthologue of *Lr1*. Regarding *Lr34* and *Lr67*, there is no report of orthologues in *Aegilops* and the results of the present study can be considered the first report of their putative orthologues. In the present study, a putative orthologue of *Lr34* showed higher expression in AT349 while those of *Lr1* and *Lr67* showed higher expression in AT350 despite showing a high IT. Though *Lr67* cannot be detected at the seedling stage, it is a hexose transporter with pleiotropic effects^70^ and its contribution to sugar metabolic process at 24 hpi may demonstrate its up-regulation. In contrary to Ling et al.^71^, *Lr1* orthologue was also detected in the susceptible accession which needs further investigation in future studies.

In plants, the majority of the cloned resistance (R) genes code for proteins with nucleotide-binding and leucine-rich repeat (NLR) domains^46^. In the present study, 46 DEGs encoding LRR motif were identified, of which 25 were up-regulated in AT349 upon infection such as ‘*AET5Gv20023600*’ with oxidoreductase activity and ‘*AET2Gv20853300*’ with lyase activity as well as ‘*AET1Gv20718200*’ encoding a protein kinase with ATP-binding function. These genes represent potential candidates for further studies against wheat leaf rust. In addition, the ‘*AET5Gv21162800*’ gene encoding NLRs domains was down-regulated in the RT_ST comparison. Although NLRs detect the presence of effector molecules and are involved in ETI, the actual immune response of plants depends on ETI interaction with many other components^47^. Yadav et al.^18^ identified 15 and 66 NLRs encoding transcripts in the compatible and incompatible interactions, respectively, and stated that leaf rust with suppression or changes of upstream signaling in the host, has likely resulted in the down-regulation of immune response and pathogen proliferation in the susceptible genotype^18^.

## Conclusion

The results of RNA seq in *Ae. tauschii* presented a comprehensive view of the incompatible and compatible response to *P. triticina* in this species. Transcriptome profiling identified significant DEGs in response to LR and exhibited implicit GO terms and/or pathways in the host–pathogen interactions, such as chromatin remodeling. This remodeling as a logistic action to organize the genome, suggests the dynamic regulation of genes to make the incompatible *Ae. tauschii* ready for sophisticated and timely defense responses to leaf rust. The other overlooked pathway was the ‘RNA degradation mechanism’, which likely accelerated the true defense responses, cellular homeostasis, and enhanced the stability of biological processes against rust. Despite all feasible defense strategies, some rust effector proteins may penetrate the leaves. However, the resistant *Ae. tauschii* accession apparently down-regulated the galactosylation activity to prevent rust colonization. Focus on such crucial pathways together with associated DEGs is suggested for future disease resistance studies, which help discover novel and putative aspects of defense mechanisms. Since a number of down-regulated DEGs associated with the susceptible phenotype were related to ‘photosynthesis’, the photosynthesis rate could be suggested as a likely physiological marker for screening different genotypes. Overall, *Ae. tauschii* as the common ancestor of wheat is a valuable genetic resource for breeding programs. From this aspect, dozens of highly significant DEGs were identified, serving as a valuable repository of candidate genes, which may aid in the development of rust resistant wheat genotypes.

## Material and methods

### Plant material and *P. triticina* pathotype

Two resistant and susceptible *Ae. tauschii* accessions*,* AT349 (TN-01-1970) and AT350 (TN-01-2017), respectively*,* were selected from a collection of wheat wild relatives collected from the southern shores of the Caspian Sea (Iran) according to the relevant guidelines and legislations or obtained from the Seed and Plant Improvement Institute (Karaj, Iran) and tested with an array of 10 *P. triticina* pathotypes^72^. The two above-mentioned accessions were obtained from the National Plant Gene Bank, Seed and Plant Improvement Institute, Iran and inoculated with pathotype CDHLQ (race No. 15 and the virulence pattern: *3a*, *3bg*, *11*, *14b*, *24*, *30*, *B*^73^) which was first multiplied on the susceptible wheat cultivar ‘Boolani’ in greenhouse. The urediniospores were mixed with talcum powder in a ratio of 1:3 and transferred onto the leaves using a fine paintbrush at the two-leaf stage. The inoculated plants were then kept in a dark and cool room (18 °C and 100% humidity) for 24 h after which were moved to rooms maintained at 18–20 °C.

### Sampling and phenotyping

Seeds of the resistant and susceptible accessions were planted in two sets of six pots. The growing conditions and inoculation procedure were the same as those mentioned earlier. One set was inoculated with the above-mentioned pathotype while the other set was kept as mock (the inoculum included only talcum powder). Leaf tissues were collected from treated and non-treated plants 24 hpi in four biological replicates as suggested by Coram et al.^74^ and Chandra et al.^58^. The samples were named resistant control (RC), resistant treatment (RT), susceptible control (SC), and susceptible treatment (ST). One biological replication was used for RNA extraction and sequencing, and the other three were analyzed by quantitative polymerase chain reaction (RT-qPCR).

Both inoculated and mock-inoculated seedlings were kept for 2 weeks to monitor disease development where plants with infection types (ITs) less than ‘2’ were considered resistant while those with ITs higher than ‘3’ were regarded as susceptible^75^.

### RNA extraction, sequencing and mapping to the reference genome

The leaf samples were ground into a fine powder in liquid nitrogen in a mortar and pestle to isolate RNA using the Qiagen RNA Isolation Kit (Germany) following the manufacturer’s protocol. The quality of RNA was determined on 1% agarose gel, and RNA concentration was measured using an Epoch microplate spectrophotometer (BioTek Instruments, Vermont, USA) at 260 nm and 280 nm. High-quality RNA samples were delivered for sequencing (Macrogen Inc., Seoul, South Korea) and total RNA integrity was checked using an Agilent Technologies 2100 Bioanalyzer (Agilent Technologies, Santa Clara, CA, USA) with an RNA Integrity Number (RIN). Then, libraries were constructed using the TruSeq RNA Library Prep Kit with insert sizes ranging from 266 to 297 bp and sequenced using the high-throughput sequencing system; Illumina HiSeq2500 where high-quality paired-end reads of 101 bp were generated. The quality of raw reads was checked with FastQC 0.11.8 (https://www.bioinformatics.babraham.ac.uk/projects/fastqc/), and reads were cleaned with Trimmomatic^76^. The ribosomal RNA (rRNA) contamination was precisely determined by SortMeRNA 2.1^77^ and filtered out. Gene model annotations of *Ae. tauschii* and *P. triticina* were obtained from the Ensembl database (https://ensemblgenomes.org/). Subsequently, the contaminant reference genome of *P. triticina* (GCA_000151525.1^78^) was identified and removed via raw data pre-alignment using ultrafast universal RNA-seq aligner, STAR 2.6.1d^79^. Finally, the remaining transcripts were aligned to the *Ae. tauschii* reference genome (Aet_v4.0, GCA_002575655.1^80^), and the transcriptome was constructed via STAR under the Linux operating system using a super computer and forwarded for downstream RNA-seq analysis.

### Annotation, normalization of gene expression levels and detection of differentially expressed genes

Reads were counted using the HTSeq 0.11.1 package^81^ in Python based on the Ensembl gene model annotation. The HTSeq package provides the utility “htseq-count” for counting reads that are mapped to each feature for a given dataset. The GTFtools 0.6.5 package^82^ was executed by the Python Interpreter to calculate the median of gene length in the *Aegilops* genome. Further, counts per million mapped reads (CPM) were normalized using the trimmed mean of M-values (TMM) approach in the edgeR package^83^ in R as a normalization method for between-sample (library) comparisons, while the FPKM normalization (Fragments Per Kilo-base per Million reads mapped) method was performed for within-sample comparisons according to the following formula:

1 $$\text{FPKM }(\text{A})= \frac{{10}^{6}\text{C}}{NL/{10}^{3}}$$

where, FPKM (A) is the expression of gene A, C is the number of fragments mapped to gene A, N is the total number of fragments mapped to the reference genes, and L is the number of bases of gene A (Gene length^84^). To demonstrate the differences between samples, principal component analysis (PCA) was performed using the samples’ expression profiling in the Factoextra R package (http://www.sthda.com/english/rpkgs/factoextra).

Differentially expressed genes between samples were identified by the NOISeq package^20^ in R using TMM normalized counts. Counts equal to zero were replaced by 0.5 and the percentage of the total sequencing depth (pnr) was considered 0.2. Comparative analysis of DEGs was performed in four levels between samples, including: resistant (AT349) treatment versus resistant control (RT_RC), susceptible (AT350) treatment versus susceptible control (ST_SC), resistant treatment versus susceptible treatment (RT_ST) and resistant control versus susceptible control (RC_SC), which are referred to as the four comparisons in this study.

### GO terms and KEGG pathway enrichment

Gene ontology (GO) analysis was performed using the biomaRt package in R to identify the main biological phenomena of DEGs; molecular function (MF), cellular component (CC), and biological process (BP). Furthermore, the GO term enrichment was realized by the classic algorithm of Fisher statistics under the topGo package in R where top enriched GOs with *P*-values less than 0.01 were selected^85^. The Kyoto Encyclopedia of Genes and Genomes (KEGG) database was used to identify the main pathways significantly enriched in DEGs^86^. The pathways with adjusted *P-*values based on a false discovery rate (FDR) less than 0.05 were defined as significantly enriched.

### Transcription factor (TF) discovery, protein domain enrichment analysis and quest for leaf rust (LR) resistance genes

The DEGs encoding TFs were identified by querying the Plant Transcription Factor Database (http://planttfdb.gao-lab.org/). In addition, a hidden Markov model (HMM) approach was implemented to search for the protein domains obtained from Pfam to scan the DEGs using the hmmscan program of the HMMER suite^87^. To identify NBS-LRR genes in the RT_RC and RT_ST comparisons, protein sequences were analyzed using InterProScan 5 standalone^88^, including all 13 integrated databases (CATH-Gene3D, HAMAP, PANTHER, PIRSF, PRINTS, PROSITE patterns, PROSITE profiles, Pfam, PfamB, Pro-Dom, SMART, SUPPERFAMILY, and TIGRFAMs) on Linux. Also, to further predict the leucine-rich repeat (LRR) motifs, the protein sequences of the DEGs were subjected to LRRsearch (http://www.lrrsearch.com). This web-server has been developed using position specific scoring matrix (PSSM) of 11 residue LRR–HCS (highly conserved segment) which are frequently observed motifs in the most divergent classes of LRR containing proteins. Furthermore, available sequences of LR resistance genes were retrieved from the NCBI database in *T. aestivum*. Then, reciprocal blast was conducted in BLASTN 2.6.0+^89^ to identify their putative orthologues in *Ae. tauschii*.

### RT–qPCR and data validation

Total RNA (2 μg) from three biological replicates was treated with RNase–free DNase I (1 U µL^−1^) 1000U (Fermentas, USA) to eliminate possible genomic contamination. Afterwards, it was reverse transcribed into cDNA using a RevertAid First Strand cDNA Synthesis Kit (TaKaRa, Japan). The qRT-PCR primers were designed using Primer3Plus (https://primer3plus.com/primer3web/primer3web_input.htm; Table S3). Then, their secondary structures were checked using Beacon Designer (http://www.premierbiosoft.com/qOligo/Oligo.jsp?PID=1) and the secondary structure of the amplicon was verified using Mfold DNA (http://unafold.rna.albany.edu/?q=mfold/DNA-Folding-Form). Finally, primers’ specificities were confirmed using NCBI primer BLAST (https://www.ncbi.nlm.nih.gov/tools/primer–blast/index.cgi?LINK_LOC=BlastHome).

RT- qPCR was performed on an ABI StepOne real-time PCR system (Applied Biosystems, USA) using the SYBR Green qPCR Master Mix (Ampliqon, Denmark) according to the manufacturer’s instructions. The genes *GAPDH* and *TUBβ* were used as housekeeping, and the average of their Ct values (Ct reference) was used for further calculations. Two technical replicates were used for each biological sample, and PCR conditions were determined based on the quality, concentration, and purity of RNA (Supplementary Tables S4 and S5). Relative quantification was calculated by the comparative 2^–ΔΔCT^ method using the following equation:

2 $${\text{Expression}}\;{\text{Ratio}}{:} \; \, 2^{{{-}\Delta \Delta {\text{CT}}}}$$

$$\Delta \Delta {\text{CT }} = \, \left( {{\text{CT}}_{{{\text{target}}}} {-}{\text{ CT}}_{{{\text{Reference}}}} } \right){\text{ Time x }}{-} \, \left( {{\text{CT}}_{{{\text{target}}}} {-}{\text{ CT}}_{{{\text{Reference}}}} } \right){\text{ Time }}0$$

where Time x is the treatment condition and Time 0 denotes the control condition^90^. Statistical significance was assessed using Student’s *t*-test (*p* ≤ 0.05). Correlation analysis was performed to determine the relationship between RNA–seq and qRT–PCR expression data.

## Supplementary Information

Supplementary Information.

## Data Availability

RNA sequencing data were deposited at the National Center for Biotechnology Information (NCBI) in the Sequence Read Archive (SRA) under the PRJNA748580 Bioproject accession.
