# Supplementary material for: Comparative transcriptome analysis of two contrasting resistant and susceptible Aegilops tauschii accessions to wheat leaf rust (Puccinia triticina) using RNA-sequencing
Source: Sci Rep. 2022 Jan 17;12:821. doi: 10.1038/s41598-021-04329-x (PMC8764039; doi:10.1038/s41598-021-04329-x)
Supplement: Supplementary file 1 — Supplementary Information. [file 41598_2021_4329_MOESM1_ESM.docx]

**Scientific Reports Journal**

**Comparative transcriptome analysis of two contrasting resistant and susceptible *Aegilops tauschii* accessions to wheat leaf rust (*Puccinia triticina*) using RNA-Sequencing**

Saeideh Dorostkar^1^, Ali Dadkhodaie^1*^, Esmaeil Ebrahimi^2,3,4^, Bahram Heidari^1^, Mahmood Ahmadi-Kordshooli^1^

1. Department of Plant Production and Genetics, School of Agriculture, Shiraz University, Shiraz, Iran
2. La Trobe Genomics Research Platform, School of Life Sciences, College of Science, Health and Engineering, La Trobe University, Melbourne, VIC 3086, Australia
3. School of Animal and Veterinary Sciences, The University of Adelaide, Adelaide, SA 5371, Australia
4. School of BioSciences, The University of Melbourne, Melbourne, VIC 3052, Australia

*Corresponding author: [dadkhodaie@shirazu.ac.ir](mailto:dadkhodaie@shirazu.ac.ir)

**Supplementary** **Table S1**. List of LRR motif-containing DEGs in RT_RC and RT_ST comparisons.

| **Gene ID** | **Comparison** | **Up/Down regulated** | **Gene ID** | **Comparison** | **Up/Down regulated** |
| --- | --- | --- | --- | --- | --- |
| *AET5Gv20023600* | RT_RC | Upregulated | *AET4Gv20005700* | RT_ST | Upregulated |
| *AET2Gv20853300* | RT_RC | Upregulated | *AET3Gv20781600* | RT_ST | Upregulated |
| *AET2Gv20481900* | RT_ST | Upregulated | *AET2Gv21032800* | RT_ST | Upregulated |
| *AET1Gv20702500* | RT_ST | Upregulated | *AET5Gv20225500* | RT_ST | Upregulated |
| *AET2Gv20036700* | RT_ST | Upregulated | *AET7Gv20736800* | RT_ST | Upregulated |
| *AET7Gv21063800* | RT_ST | Upregulated | *AET6Gv20761300* | RT_ST | Upregulated |
| *AET6Gv20210900* | RT_ST | Upregulated | *AET6Gv20414400* | RT_ST | Upregulated |
| *AET4Gv20650200* | RT_ST | Upregulated | *AET4Gv20539600* | RT_ST | Upregulated |
| *AET1Gv20787800* | RT_ST | Upregulated | *AET6Gv20292900* | RT_ST | Down regulated |
| *AET6Gv20041500* | RT_ST | Upregulated | *AET5Gv21162800^*^* | RT_ST | Down regulated |
| *AET3Gv20747600* | RT_ST | Upregulated | *AET6Gv20506000* | RT_ST | Down regulated |
| *AET4Gv20184600* | RT_ST | Upregulated | *AET2Gv20767900* | RT_ST | Down regulated |
| *AET1Gv20718200* | RT_ST | Upregulated | *AET7Gv20103100* | RT_ST | Down regulated |
| *AET1Gv20996400* | RT_ST | Upregulated | *AET1Gv20204300* | RT_ST | Down regulated |
| *AET3Gv20611300* | RT_ST | Upregulated | *AET5Gv20099700* | RT_ST | Down regulated |
| *AET2Gv21044900* | RT_ST | Upregulated | *AET7Gv20648500* | RT_ST | Down regulated |
| *AET4Gv20128500* | RT_ST | Upregulated | *AET6Gv20292700* | RT_ST | Down regulated |
| *AET5Gv20604300* | RT_ST | Upregulated | *AET6Gv21012400* | RT_ST | Down regulated |
| *AET2Gv20220100* | RT_ST | Upregulated | *AET6Gv20395200* | RT_ST | Down regulated |
| *AET1Gv20942900* | RT_ST | Upregulated | *AET5Gv21060200* | RT_ST | Down regulated |
| *AET6Gv20787600* | RT_ST | Upregulated | *AET7Gv21190000* | RT_ST | Down regulated |
| *AET1Gv20789200* | RT_ST | Upregulated | *AET2Gv20769500* | RT_ST | Down regulated |
| *AET5Gv21180100* | RT_ST | Upregulated |  |  |  |

Note: * indicates NB-LRR domain, RC (resistant control), RT (resistant treatment) and ST (susceptible treatment).

**Supplementary Table S2**. Summary of putative orthologues of leaf rust resistance genes in *Aegilops tauschii*. The orthologues identified using reciprocal blast in BLASTN 2.6.0+.

| Query (*Triticuum aestivum*) | GenBank (*T. aestivum*) | CDS (*T. aestivum*) | Length of query | Putative orthologues in *Ae. tauschii* | Length of putative orthologues | Coverage | Identity percentage |
| --- | --- | --- | --- | --- | --- | --- | --- |
| *Lr1* | EF439840.1 | ABS29034.1 | 4035 | *AET5Gv21241000* | 5014 | 3969/4035 | 98 % |
| *Lr22a* | KY064064.1 | KY064064.1 | 2739 | *AET2Gv20074800* | 3477 | 2684/2741 | 98 % |
| *Lr34*(1)* | FJ436983.1 | ACN41353.1 | 1539 | *AET7Gv20224400* | 1565 | 1539/1539 | 100 % |
| *Lr34*(2) | FJ436983.1 | ACN41354.1 | 4206 | *AET7Gv20224500* | 5641 | 4204/4209 | 99 % |
| *Lr34*(3) | FJ436983.1 | ACN41355.1 | 1554 | *AET7Gv20224900* | 1819 | 1554/1554 | 100 % |
| *Lr34*(4) | FJ436983.1 | ACN41356.1 | 2022 | *AET7Gv20225000* | 2353 | 1965/2022 | 97 % |
| *Lr34*(5) | FJ436983.1 | ACN41357.1 | 2031 | *AET7Gv20225100* | 2203 | 1960/2027 | 97 % |
| *Lr34*(6) | FJ436983.1 | ACN41358.1 | 1557 | *AET7Gv20225200* | 1910 | 1526/1557 | 98 % |
| *Lr67* | MK425206.1 | QEA08561.1 | 1545 | *AET4Gv20606400* | 2074 | 1543/1545 | 99 % |
| Note: * numbers 1-6 in parentheses show different isoforms of *Lr34*. | | | | | | | |

**Supplementary Table S3.** Specific primers used to measure the expression of 10 DEGs by RT-qPCR in the resistant and susceptible *Aegilops tauschii* accessions. The RT-qPCR primers were designed using Primer3Plus (<https://primer3plus.com/primer3web/primer3web_input.htm>).

| **Groups** |  | **Gene accession** | **Forward primer 5'–3'** | **Reverse primer 5'–3'** |
| --- | --- | --- | --- | --- |
| **RT_RC comparison** | Up-regulated DEGs | AET2Gv20465900 | CAGGATGTGATGAAGGTTGAGAAGG | AAAGTATCCATTTCCAGAGCTGATGT |
|  |  | AET5Gv20023600 | TAAGCTGAGTTGTTTGTGTGTGCGT | TGTCCCATTCCAATTATACCCACCGT |
|  | Down-regulated DEGs | AET2Gv20945400 | TGAAACACATCCATCTATCTGCTCC | AAATGCTCTACTACTGGCTTCTTCC |
|  |  | AET2Gv21186600 | GCAACTTTGTGAATTGTTTAGTGGTGGG | TACAGAAGAAACAGCAGGGAGGGAG |
| **ST_SC comparison** | Down-regulated DEGs | AET1Gv20065900 | GTCTTCATCCCGCTATGCCGAAAC | CATGCCGATGATGGACAGGACC |
|  |  | AET7Gv21176900 | GACATTGCCACTGTGATCCCCAAC | TTCTCAACTACAAGCAAGCCCGAGT |
|  |  | AET6Gv20762000 | TCTTCAGTCTTCAGTGGCAAATTGTG | TTTCGATTGGCATAGCAAGGAATGG |
|  | Up-regulated DEGs | AET2Gv20624400 | TGGTTTTGTGCTGTTGTTGTTACTCG | TGAACCACGCTCTTTATTTTGTAGTTGC |
|  |  | AET7Gv21322900 | CTACTTGATGCTTCCTGTGAACGCC | ACCTCCTCATCCCTGTGCATGAAAT |
| **RT_ST comparison** | Up-regulated DEGs | AET7Gv20865000 | CATCAACACGAAAGCAATTCTACCAA | CCTCTTAGGACACAAACAAGACGAA |
| **Housekeeping genes** | ––– | *GAPDH* | TGTCCATGCCATGACTGCAA | CCAAGTGCTGCTTGGAATGATG |
|  |  | *TUBβ* | CAAGGAGGTGGACGAGCAGATG | GACTTGACGTTGTTGGGGATCCA |
| **Note:** RC (Resistant Control), RT (Resistant Treatment), SC (Susceptible Control), ST (Susceptible Treatment), DEGs (differentially expressed genes), GAPDH (Glyceraldehyde–3–Phosphate Dehydrogenase) and TUBβ (tubulin beta). | | | | |

**Supplementary Table S4.** PCR conditions for differentially expressed genes (DEGs) in transcriptome analysis of the resistant and susceptible *Aegilops tauschii* accessions.

| **PCR step** | **Temperature** $\boldsymbol{℃}$ | **Protocol runs**  **(Minutes)** | **Cycle** |
| --- | --- | --- | --- |
| Initial denaturation | 95 | 5 | 1 |
| Denaturation | 95 | 1 | 35 |
| Annealing | Primer Temperature | 1 |  |
| Extension | 72 | 1 |  |
| Final extension | 72 | 5 | 1 |

**Supplementary Table S5.** RT-qPCR conditions for differentially expressed genes (DEGs) in transcriptome analysis of the resistant and susceptible *Aegilops tauschii* accessions.

| PCR cycling step | | Number of cycles | Temperature $℃$ | Protocol runs  (Minutes) |
| --- | --- | --- | --- | --- |
| Initial denaturation | | 1 | 95 | 5 |
| Denaturation | | 40 | 95 | 1 |
| Annealing | AET2Gv20465900 |  | 59 | 1 |
|  | AET5Gv20023600 |  | 62 |  |
|  | AET2Gv20945400 |  | 59 |  |
|  | AET2Gv21186600 |  | 62 |  |
|  | AET1Gv20065900 |  | 61.5 |  |
|  | AET6Gv20762000 |  | 60 |  |
|  | AET7Gv21176900 |  | 62 |  |
|  | AET2Gv20624400 |  | 61 |  |
|  | AET7Gv21322900 |  | 62 |  |
|  | AET7Gv20865000 |  | 59 |  |
|  | *GAPDH* |  | 59 |  |
|  | *TUBβ* |  | 58 |  |
| Extension | |  | 72 | 5 |
| Final extension | | | 72 | 5 |


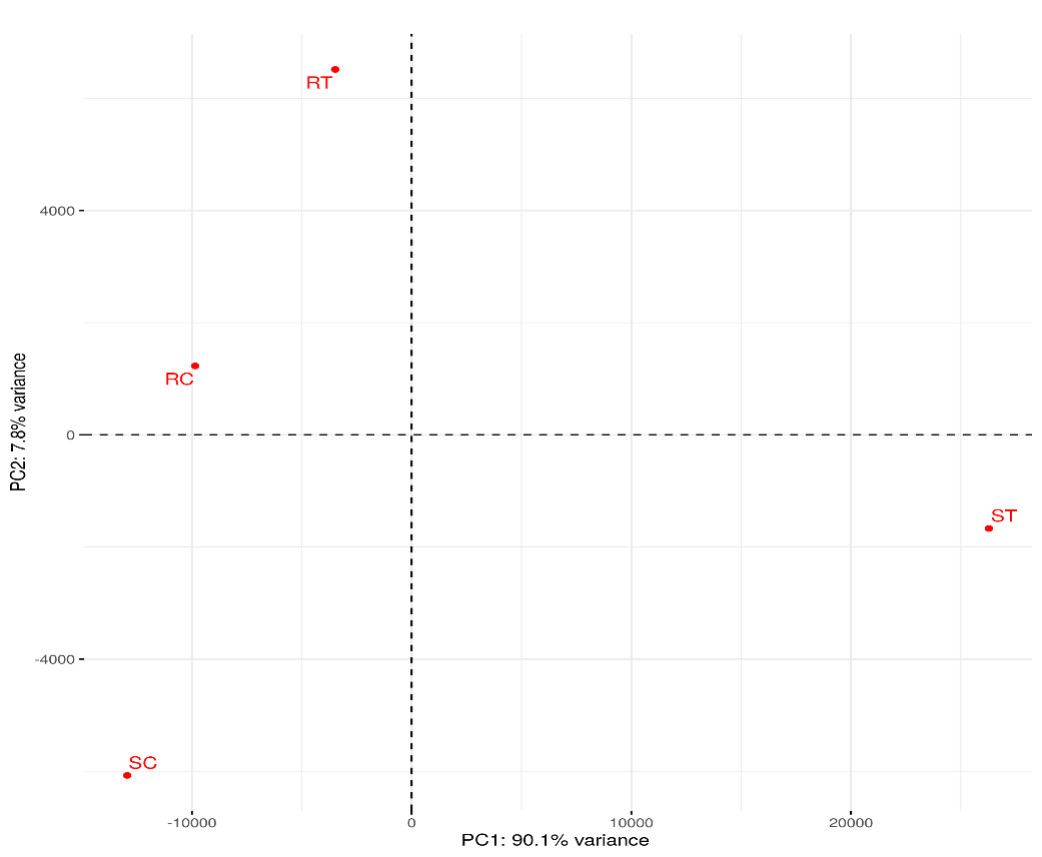


**Supplementary Fig. S1.** Principal component analysis (PCA) of *Aegilops tauschii* samples. The x and y axes represent the two principal components of the total variance, 90.1% and 7.8%, respectively. Each dot represents RNA samples subjected to control and infected conditions. RC (resistant control), RT (resistant treatment, at 24 hpi), SC (susceptible control), and ST (susceptible treatment, at 24 hpi). The plot was created using ‘ggplot2 version 3.3.5’ R/CRAN package (<https://ggplot2.tidyverse.org>).

**
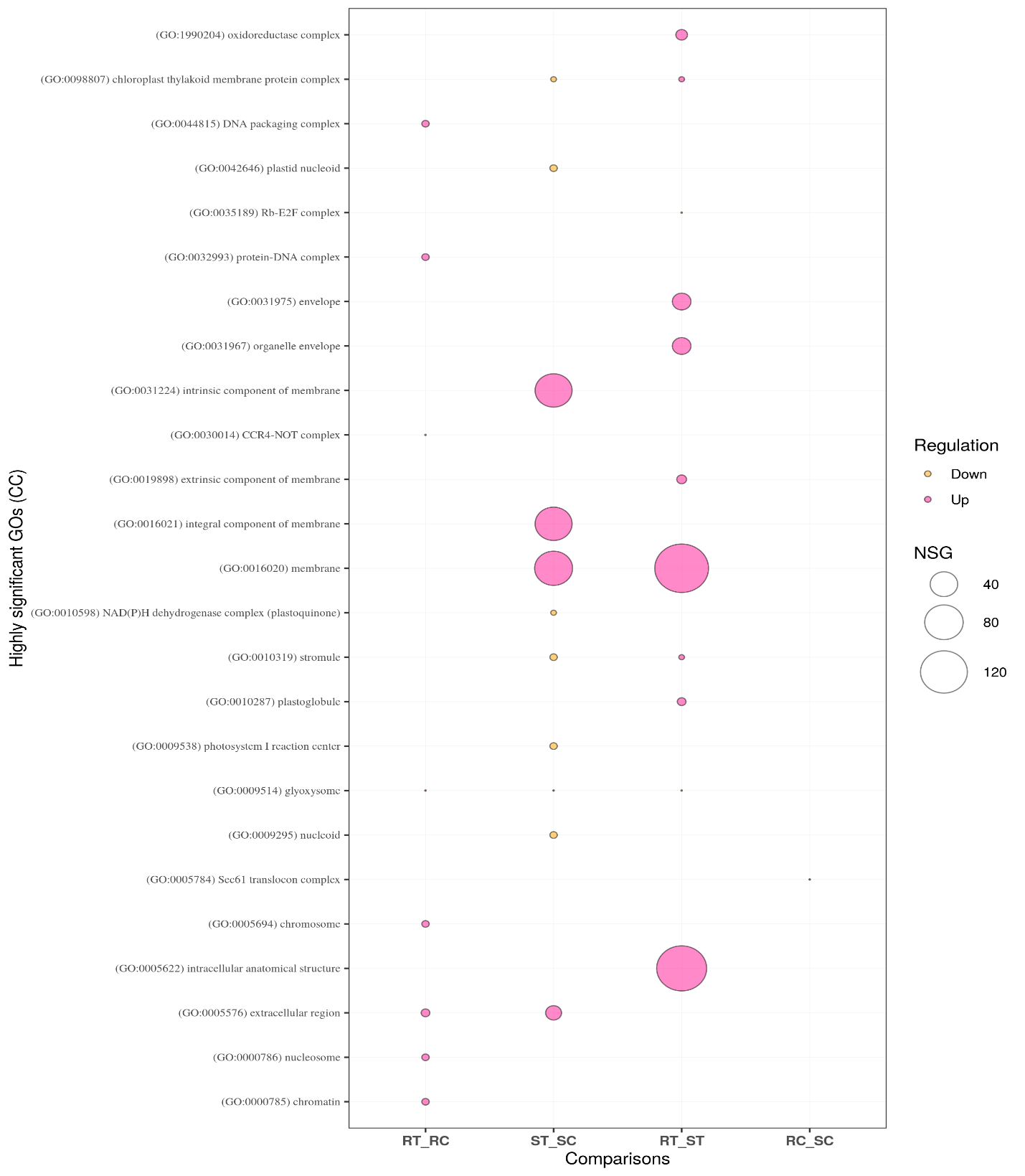
**

**Supplementary Fig. S2.** Bubble plot of GO enrichment terms related to cellular component (CC). NSG: The size of the circles shows the number of significant genes associated with each GO term. Purple and orange colors show up-regulated and down-regulated DEGs, respectively. RC (resistant control), RT (resistant treatment), SC (susceptible control), and ST (susceptible treatment). The plot was created using ‘ggplot2 version 3.3.5’ R/CRAN package (<https://ggplot2.tidyverse.org>).


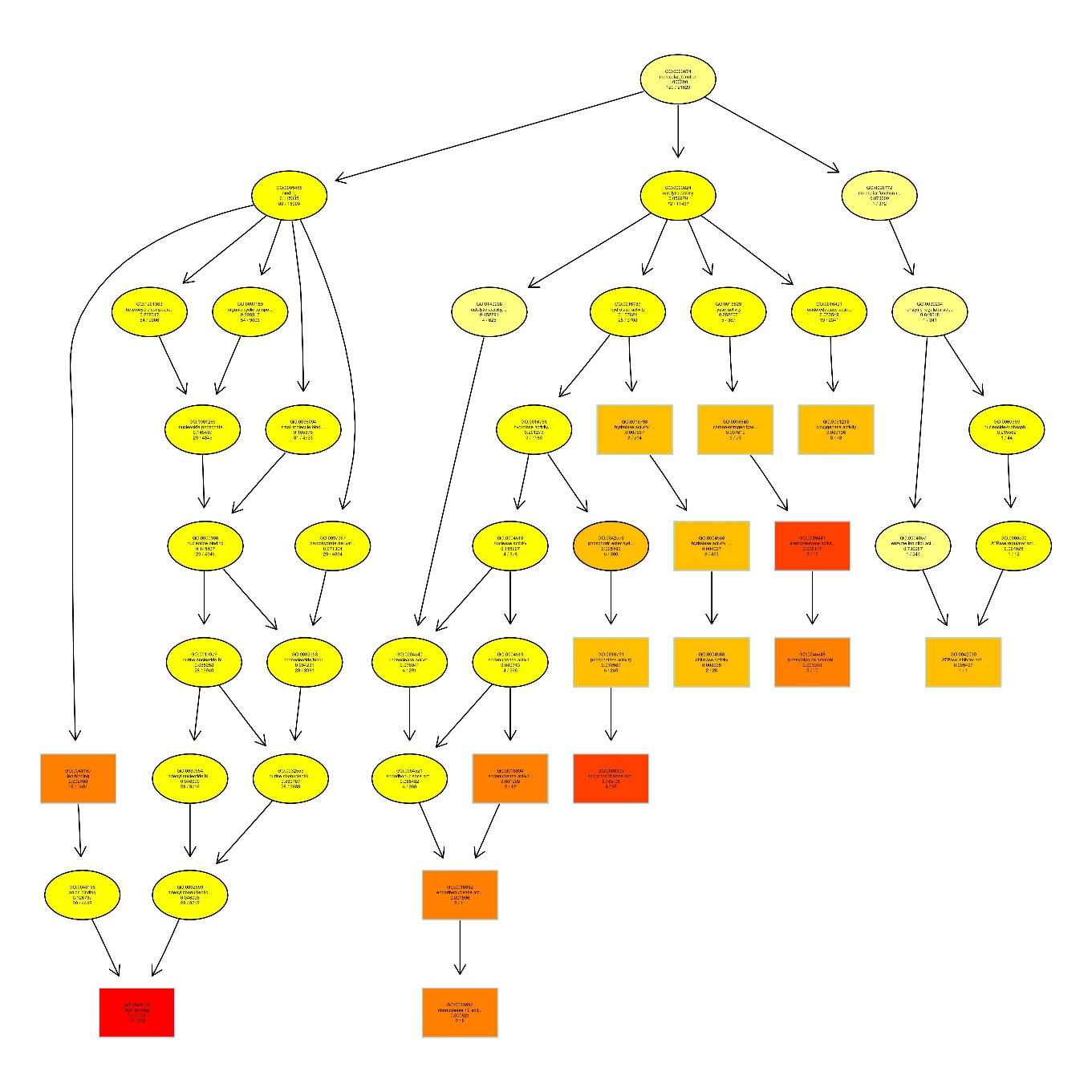


**Supplementary Fig. S3**. Schematic graph of molecular function of the up-regulated DEGs in the RC_SC comparison. Rectangles denote 15 most significant GO terms. Dark red and the bright yellow colors represent the most and the least significant GOs, respectively. RC (resistant control) and SC (susceptible control).The graph was created using ‘topGo’ R/Bioconductor package [83].


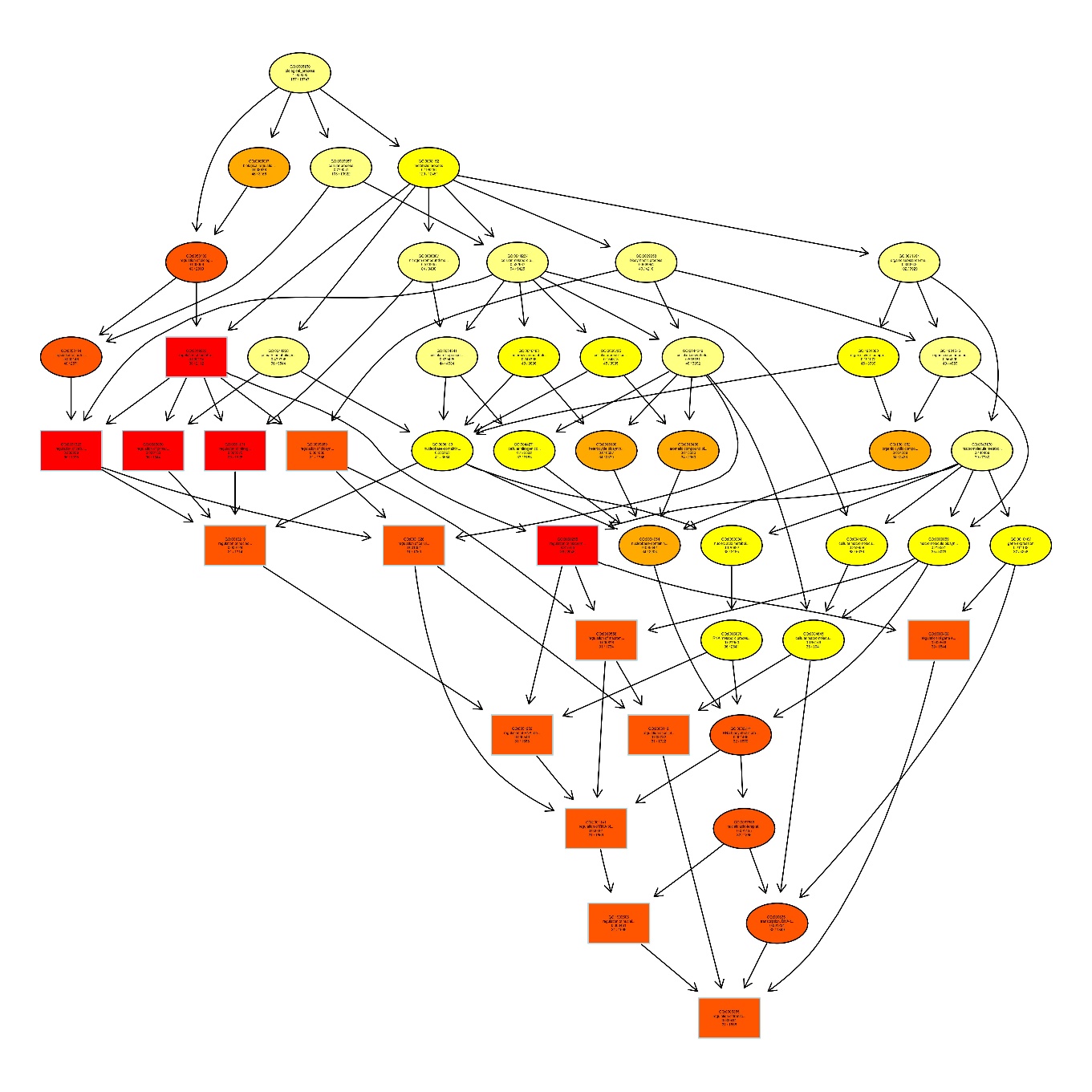


**Supplementary Fig. S4.** The schematic graph of 15 top BP-GO terms enriched by classic algorithm of Fisher statistics in the RT_ST comparison. Rectangles denote 15 most significant GO terms. Dark red and bright yellow colors represent the most and the least significant GOs, respectively. RT (resistant treatment) and ST (susceptible treatment). The graph was created using ‘topGo’ R/Bioconductor package [83].
